# Biomaterial-based strategies for bone cement: modulating the bone microenvironment and promoting regeneration

**DOI:** 10.1186/s12951-025-03363-5

**Published:** 2025-05-13

**Authors:** Jiawei Jiang, Juan Wang, Pan Fan, Zhe Zhao, Hongjian Deng, Jian Li, Yi Wang, Yuntao Wang

**Affiliations:** 1 Medical School of Southeast University, Nanjing, 210009 Jiangsu China; 2 Department of Spine Center, Zhongda Hospital, Southeast University, Nanjing, 210009 Jiangsu China; 3 Department of Orthopaedics, Jiujiang Traditional Chinese Medicine Hospital, Jiujiang, 332000 Jiangxi China; 4 Central Laboratory, Gaochun Hospital Affiliated to Jiangsu University, Nanjing, 211300 Jiangsu China; 5 Department of Orthopaedics, Xuyi People’s Hospital, Xuyi, 211700 Jiangsu China; 6 Department of Orthopaedics, The Affiliated 2 Hospital of Nantong University, Nantong, 226001 Jiangsu China

**Keywords:** Bone cement, Biomaterials modification, Bone regeneration, Osteoporosis, Bone microenvironment

## Abstract

Osteoporotic bone defect and fracture healing remain significant challenges in clinical practice. While traditional therapeutic approaches provide some regulation of bone homeostasis, they often present limitations and adverse effects. In orthopedic procedures, bone cement serves as a crucial material for stabilizing osteoporotic bone and securing implants. However, with the exception of magnesium phosphate cement, most cement variants lack substantial bone regenerative properties. Recent developments in biomaterial science have opened new avenues for enhancing bone cement functionality through innovative modifications. These advanced materials demonstrate promising capabilities in modulating the bone microenvironment through their distinct physicochemical properties. This review provides a systematic analysis of contemporary biomaterial-based modifications of bone cement, focusing on their influence on the bone healing microenvironment. The discussion begins with an examination of bone microenvironment pathology, followed by an evaluation of various biomaterial modifications and their effects on cement properties. The review then explores regulatory strategies targeting specific microenvironmental elements, including inflammatory response, oxidative stress, osteoblast-osteoclast homeostasis, vascular network formation, and osteocyte-mediated processes. The concluding section addresses current technical challenges and emerging research directions, providing insights for the development of next-generation biomaterials with enhanced functionality and therapeutic potential.

**Graphical Abstract:**

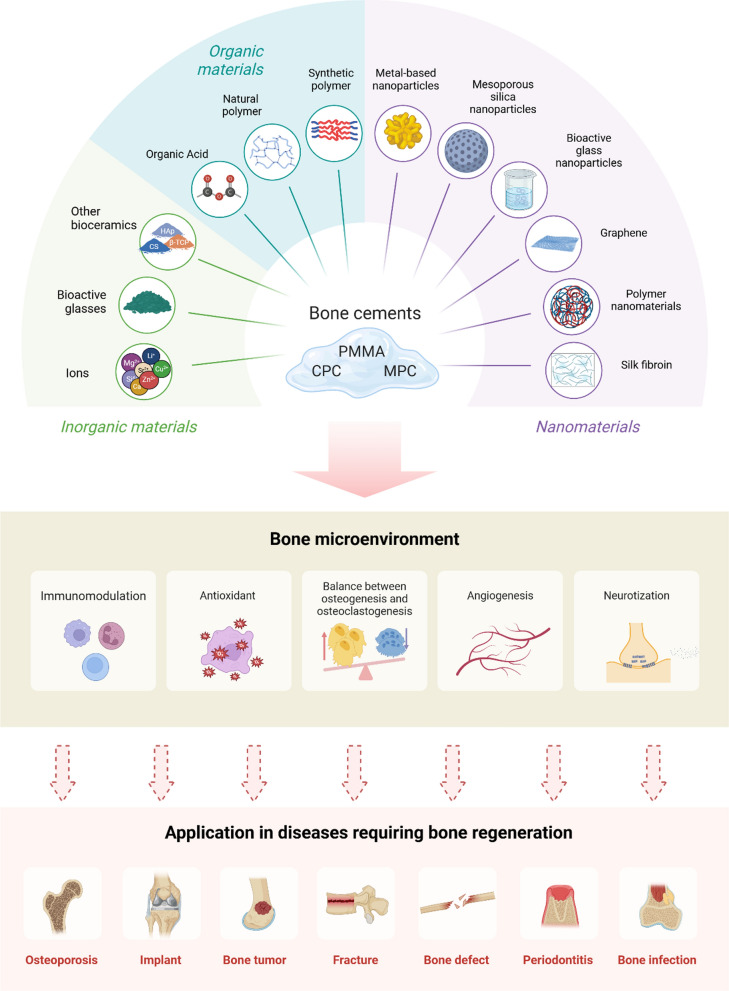

## Introduction

As the aging population increases, bone loss and decreased bone strength are common in the elderly, which usually increases the frequency of osteoporosis and leads to fractures [1]. Due to deterioration in physiology in the elderly, bone regeneration is significantly slowed, substantially decreasing the quality of life [2, 3]. Meanwhile, the inflammation also leads to defects of connective tissues and bones, like periodontitis [4]. These conditions are usually precipitated by an imbalance in bone remodeling, disruption of bone microenvironment, and bone homeostasis disorders [5, 6]. Damaged bone is more often confronted with problems such as insufficient osteoblast viability, hyperactivity of osteoclasts, vascular occlusion, local oxidative stress damage, and microbial infection [7–9]. The gold standard for bone reconstruction is still bone grafts at present which can provide osteo-inductive signals, osteogenic cells, and an osteoconductive matrix [10], but are still limited by challenges such as shortage, necrosis, and infection of bone tissue donors [11].

**Scheme 1:**
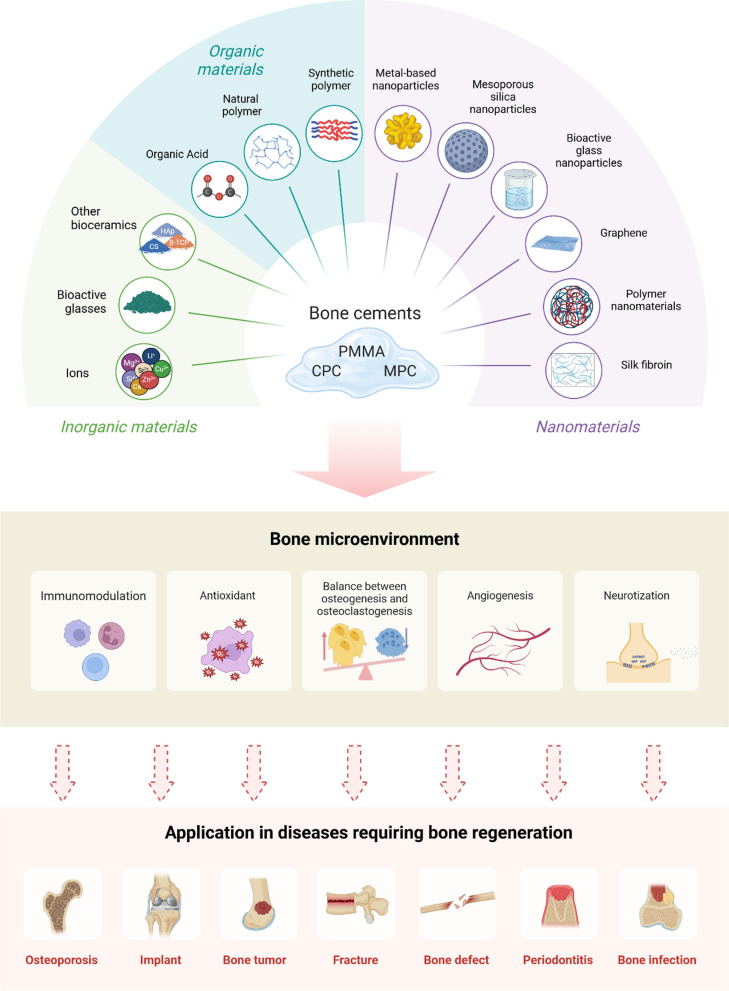
The schematic diagrams illustrate strategies for modifying bone cement using different biomaterials. These modifications regulate the bone microenvironment during different healing phases. They show broad application prospects in the research of different disease models and bone repair, etc

Bone cement (BC) has been applied extensively in repairing the bone defects caused by orthopedic trauma, osteoporosis, and periodontitis, and when used for internal fixation of artificial joint replacement for years [12]. Bone cement was first used in spinal fractures by Deramond and Galibert in 1984, and it can provide immediate stability and relieve pain [13]. Bone defects of long bones, especially the tibia and fibula would complicate bone regeneration and may lead to nonunion due to a single blood supply [14]. Masquelet et al. (2000) [15] applied bone cement to long bone fractures with bone defects greater than 5 cm. The bone cement can be easily shaped to fill irregular bone defects and surroundings and then induce the formation of membranes [16]. Infected bone defects are a common clinical conundrum in orthopedics and oral and maxillofacial surgery. Bone cement, loaded with antibiotics, has been used to control local bone infections in open fractures and repair periodontal bone [17, 18]. Antibiotic-loaded bone cement is often used in artificial joint replacement for fixing joint prostheses, and an important aspect of the success of a cemented joint replacement lies in its long-term bond between bone and cement [19, 20] (Scheme 1). However, the three bone cements that are currently in use have drawbacks. Polymethylmethacrylate (PMMA)-based bone cement faces critical limitations including high exothermic polymerization temperature, non-biodegradability, insufficient bioactivity, monomer toxicity, poor osteo-conductivity, mechanical mismatch with natural bone [21–24], potentially leading to aseptic loosening, impaired bone regeneration, adjacent tissue complications, and so on [9, 25]. Nevertheless, PMMA bone cement remains the most widely used bone cement in clinical practice owing to its superior mechanical strength (70–100 MPa) and remarkable stability. Calcium phosphate cement (CPC) attracted increasing concerns in fields of bone repair and spine fixing due to their chemical similarity to natural bone hydroxyapatite [26]. However, the application of traditional CPC is hindered by several limitations, including poor mechanical properties (compressive strength of only 5–40 MPa) [27], insufficient washout resistance [28], uncontrolled degradation rate that mismatches with new bone formation prolonged [29], and setting time (approximately 30 min) which is not convenient for surgical operations [30]. Magnesium phosphate cement (MPC) has emerged as a promising bone regeneration material due to its superior properties including high initial mechanical strength (2–45 MPa), rapid setting time, moderate degradation efficiency (7–10 months), and excellent biocompatibility, offering significant advantages over traditional calcium phosphate cement [31–33]. It has been reported that Mg^2+^ ions play an important role in bone regeneration through releasing in vivo to increase osteoblast activity [34]. Despite its advantages, MPC still faces critical challenges that limit its clinical application, including high exothermic reaction and rapid setting [35]. Although these issues can be partially addressed by using high-temperature sintered MgO and borax additives, concerns remain regarding the limited effectiveness of MgO and the potential toxicity of borax [36, 37]. Additionally, the unique skeletal-adhesive structure of MPC results in poor long-term stability during degradation [35] (Table 1). In conclusion, so far there is no one ideal bone cement that can fulfill the multiple requirements of not only maintaining appropriate reaction temperature, excellent osteo-conductivity, and superior biocompatibility for bone regeneration promotion but also providing adequate mechanical strength while exhibiting controlled degradation kinetics that match the bone regeneration process.

**Table 1:** Comparison of Different Bone Cements'Properties and Applications

Type	Mechanical Strength	Degradation Rate	Biocompatibility	Osteogenic Properties	Features	Refs.
PMMA	High(70–100 MPa)	Non-degradable	Moderate possible thermal necrosis during polymerization	Limited	High mechanical strength and stability Modifiable for improved performance Non-biodegradable Limited bioactivity without modifications	[21–25]
CPC	Moderate (5–40 MPa)	18–36 months	Excellent similar to natural bone composition	Excellent osteoconductivity; natural bone bonding	Poor load-bearing strength Good biocompatibility and osteoconduction Relatively slow degradation rate Limited osteogenic potential	[26–30]
MPC	Moderate (2–45 MPa)	7–10 months	Excellent due to Mg^2+^ being natural bone component	Good through stimulating osteoblast differentiation	High early-stage strength Fast-setting Moderate degradation rate Relatively low bioactivity	[31–35]

With the rapid development of materials science in recent years, composite materials are increasingly used in bone regeneration. In 2022, Yu et al. (2022) [38] demonstrated that hydroxyapatite nanorods with a high aspect ratio (HAp-100) enhance bone regeneration through modulating T cell-mediated immune response and IL-22 production, revealing a novel mechanism of nanomaterial-guided osteogenic. Zhang et al. (2023) [39] developed a multifunctional 3D-printed scaffold by incorporating CeO2 nanoparticles into bioactive glass, creating a therapeutic platform that combines enhanced mechanical properties, antioxidative activity, and improved osteogenic capability for effective bone tissue engineering. In 2024, Cai et al. (2024) [40] developed an engineered hierarchical hydrogel that promotes mitochondrial transfer from macrophages to Bone marrow mesenchymal stem cells (BMSCs). This immune-responsive system restored cellular bioenergy metabolism and enhanced bone regeneration in inflammatory environments. Numerous studies have demonstrated that modifying bone cements through strategic incorporation of composite materials enabled synergistic benefits of enhanced osteogenic and angiogenic properties, preserved mechanical strength, and improved biodegradation profiles, achieving promising outcomes in bone regeneration applications [41–43] (Scheme 1). However, to our best knowledge, no review has comprehensively summarized the advances of composite-modified bone cements for bone regeneration so far. Herein, we present the benefits of composite-modified bone cements for bone defect treatment and review recent advances in this field. Additionally, we discuss the promising applications of these composite-modified bone cements in orthopedic medicine and propose perspectives on bone microenvironment-driven composite design strategies to advance clinical translation.

## Factors affecting bone regeneration after bone cement filling

### Bone tissue aspect

#### Balance between osteogenesis and osteoclastogenesis

Following the initial phase of immune regulation, peri-cement bone regeneration progresses through the bone formation and remodeling phases, which are primarily orchestrated by osteoblasts and osteoclasts [44]. The maintenance of bone homeostasis relies on the balance between osteoblast-mediated bone formation and osteoclast-mediated bone resorption, coordinated through complex intercellular signaling pathways. Through the secretion of Rankl and osteoprotegerin, osteoblasts and osteocytes control osteoclast differentiation and bone resorption, while osteoclasts stimulate osteoblastic responses through direct cell-to-cell contact to couple bone formation with resorption [45–47]. Notably, magnesium ions orchestrate the balance between osteoclasts and osteoblasts by suppressing osteoclastogenesis through down-regulation of pro-inflammatory cytokines, while simultaneously creating a favorable microenvironment for osteogenic cell recruitment and bone regeneration [48].

#### Angiogenesis and neurotization

During bone defect healing, angiogenesis precedes and facilitates osteogenesis, with these processes exhibiting a complementary relationship that has been termed “angiogenic-osteogenic coupling” due to their intimate spatial and temporal connection [49–51]. Vascularization-mediated bone regeneration represents a sophisticated biological process wherein endothelial cells play pivotal roles in orchestrating both hematopoietic and osteogenic activities [52]. During bone development and repair, endothelial cells establish specialized niche microenvironments that not only support hematopoietic stem cell maintenance for blood formation but also engage in intricate crosstalk with osteoprogenitor cells [53]. This process is particularly exemplified in intramembranous ossification, where mesenchymal cells directly differentiate into osteoblasts. Furthermore, extensive research has revealed the existence of a complex neuro-osteogenic network, highlighting the critical importance of crosstalk between neural and skeletal systems during development [54, 55]. This intricate regulatory network encompasses neuro-skeletal, neurovascular, and neuroimmune interactions at various stages of bone regeneration, involving specific nerve-associated cellular and molecular mechanisms that orchestrate osteogenesis and other essential bone formation processes [56]. Given these sophisticated biological interactions, the development of innovative stratified biomimetic constructs capable of simultaneously promoting neurovascular regeneration and osteogenesis represents a promising strategy for accelerating bone regeneration.

#### Immunomodulation

The immune system, comprising both innate components (neutrophils, macrophages, and dendritic cells) and adaptive elements (T and B lymphocytes), plays a crucial role in bone regeneration, which has been extensively investigated in bone tissue engineering applications [57, 58]. Immunomodulatory approaches targeting macrophages and T cells to promote bone healing have been extensively investigated. M2 macrophage-regulated inflammation resolution is essential for bone formation, as demonstrated by impaired vascularization and osteogenesis in aged models with compromised M2 function [59]. Schlundt et al. (2018) [60] discovered that the administration of cytokines IL-4/IL-13, which facilitate M2 macrophage differentiation, enhanced ossification during fracture repair. In contrast to macrophages, the function of adaptive immune cells in skeletal regeneration remains less explored. The immune response, marked by the sequential recruitment of T and B lymphocytes, exhibits complex interactions with the osseous microenvironment after trauma [61]. CD8 + T cells show adverse effects on bone repair, however, specific interventions like Iloprost delivery through fibrin matrices can suppress their inflammatory activity and accelerate bone regeneration [62]. Certainly, research has demonstrated that appropriate modulation of the inflammatory response during the early stage significantly promotes bone regeneration. For example, neutrophils are the first to respond to injured tissue and secrete cytokines to recruit macrophages within a few hours of the injury. Meanwhile, the timely and efficient phenotypic transformation of macrophages from M1 to M2 state serves dual functions: alleviating acute inflammation through the secretion of anti-inflammatory cytokines while simultaneously releasing osteogenesis-related mediators, thereby creating a favorable osteoimmunomodulatory microenvironment for enhanced bone regeneration [44].

### Bone cement aspect

#### Osteoconductivity

Osteoconductivity, a critical property in bone regeneration, is characterized by the ability of materials to support osteoblast interaction and facilitate bone growth through their porous structure, enabling osteoprogenitor cells to migrate, proliferate, differentiate, and deposit extracellular matrix (ECM) [63]. This process is typically mediated by the formation of the thin carbonated hydroxyapatite (HAp) layer on the material surface, which promotes protein adsorption and subsequent osteoblast adhesion, leading to enhanced matrix deposition. However, conventional materials such as PMMA bone cement lack this osteoconductive property, resulting in the absence of physicochemical bonding between the bone and cement interface, thereby limiting their effectiveness in bone regeneration applications [64, 65]. CPC has been extensively applied in bone defect repair, attributed to their compositional resemblance to natural bone hydroxyapatite and remarkable biological performance in terms of biocompatibility and osteoconductivity [66]. MPC exhibits limited porosity and permeability, which impede osteogenic cell infiltration and vascularization—crucial factors for bone regeneration. However, various modifications of MPC can enhance its osteoconductivity through improved porosity, surface fiber structure, and cell adhesion. These modifications include polymers (chitosan, nanofiber) [42, 67], organic salts (citrate) [68], mesoporous silica nanoparticles (MSN) [69], and so on, which have been demonstrated to enhance the MPC's osteoconductivity properties.

#### Osteoinductivity

Osteoinduction, a fundamental biological mechanism observed in bone defect healing and implant incorporation, involves the recruitment and stimulation of immature cells to differentiate into preosteoblasts and osteoprogenitor cells during the initial week post-injury or implantation [70]. Different bone cements exhibit varying degrees of osteoinductive potential: in the Masquelet technique, PMMA implantation induces the formation of a bioactive membrane rich in growth factors, including vascular endothelial growth factor, transforming growth factor β1, and bone morphogenetic protein-2, thereby facilitating transplanted bone healing [15]. MPC demonstrates notable osteoinductivity through Mg^2+^ release, which promotes BMSCs differentiation into osteoblasts by upregulating osteogenic genes (BMP-2, Runx2, ALP, OCN) and modulating intracellular calcium homeostasis and metabolism [71], whereas CPC possesses relatively limited intrinsic osteoinductive capabilities.

#### Biocompatibility

Biocompatibility represents the harmonious interaction between a biomaterial and host tissue, characterized by the material's ability to perform its intended function without eliciting adverse local or systemic responses while maintaining appropriate biological functionality. In bone tissue engineering, various bone cements have demonstrated distinct biocompatibility profiles [72]. For example, PMMA has established itself as a functional polymer material with proven biocompatibility [73], while CPC exhibits superior tissue compatibility due to its compositional similarity to the natural bone mineral phase [74]. Similarly, the exceptional biocompatibility of MPC has been validated through regulatory recognition, as exemplified by the FDA approval in 2009 of a degradable potassium-based MPC formulation developed by Bone Solutions Incorporated (BSI) for bone defect repair applications [75]. These diverse bone cement formulations, with their respective biocompatibility characteristics, have thus become integral components in contemporary bone regeneration strategies.

#### Degradation

**Fig. 1:**
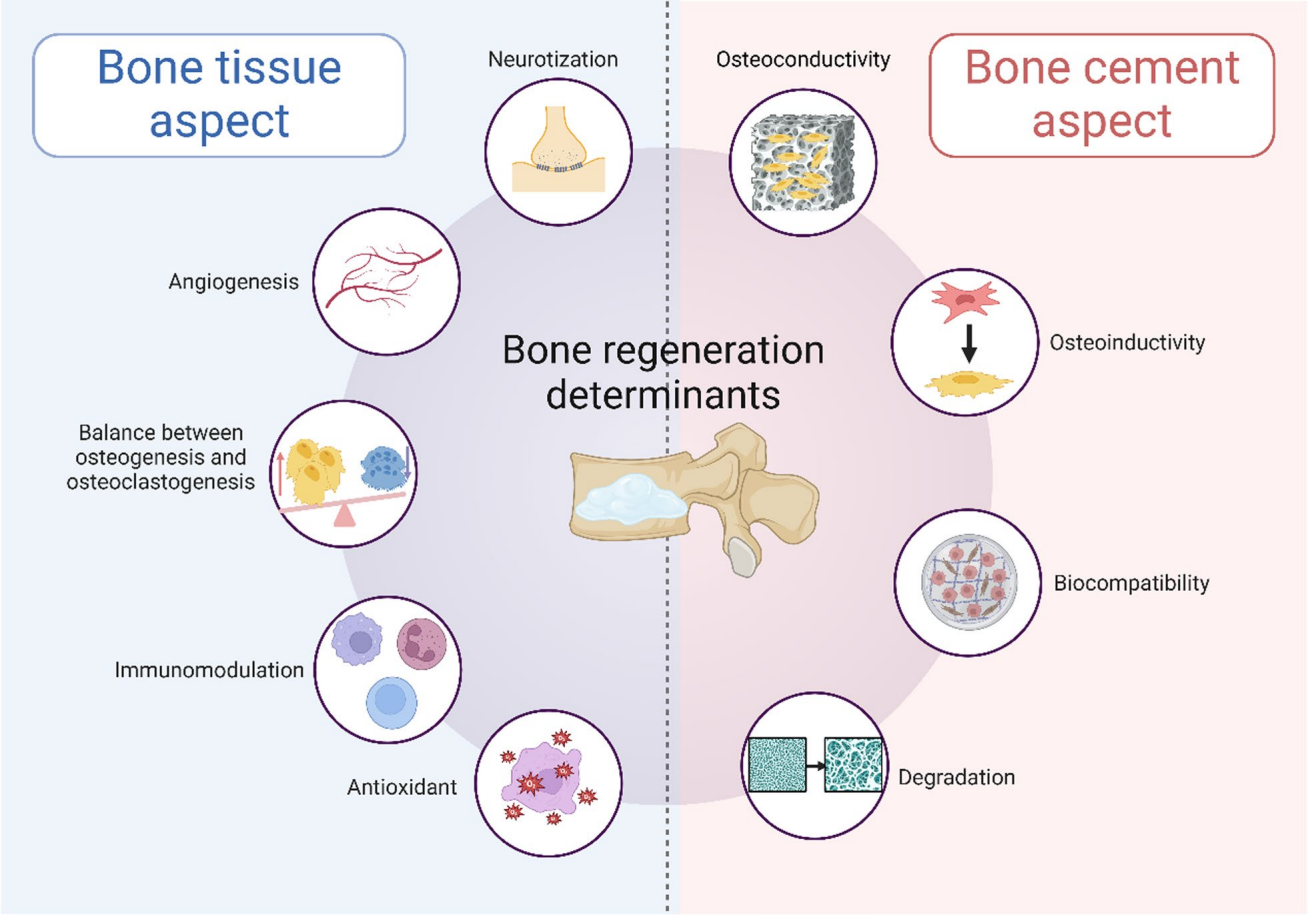
Schematic illustration of key determinants influencing bone regeneration following bone cement implantation. Created with BioRender.com

The in vivo degradation of bone cements plays a crucial role in successful bone regeneration, as appropriate degradation not only minimizes adverse physiological effects but also creates essential space for bone and tissue ingrowth [76]. However, this process requires precise temporal coordination, as premature degradation before adequate bone formation can compromise structural support, while delayed degradation may impede tissue regeneration. Among various bone cements, PMMA's non-degradable nature limits its effects on bone reconstruction, often leading to adjacent bone complications and refractures [77]. While CPC demonstrates biodegradability, its clinical utility is constrained by a slow degradation rate coupled with inherent mechanical limitations including low strength and high brittleness [78]. In contrast, MPC exhibits accelerated degradation compared to CPC, potentially facilitating enhanced bone and tissue regeneration, but this too-rapid degradation rate presents challenges in maintaining adequate mechanical support during the healing process [79] (Fig. 1).

## Applications of inorganic materials modification strategies for bone cement

Inorganic materials have garnered significant research interest due to their remarkable capacity to promote multiple tissue regeneration processes, including angiogenesis, osteogenesis, adipogenesis, wound healing, and myocardial regeneration through controlled ionic release mechanisms [80]. For example, silicon ions released from Calcium silicate (CS) bioceramics activate the bone morphogenetic protein 2 (BMP2) signaling pathway to enhance bone mineralization [81, 82]; magnesium ions orchestrate the modulation of local osteoimmune microenvironment conducive to bone regeneration; phosphate ions from phosphorus-rich materials stimulate mineralization processes [83]; and strontium ions released from Sr-substituted bioactive glass implants facilitate controlled therapeutic effects on bone formation [84]. Therefore, the strategic incorporation of inorganic materials into bone cement formulations represents a promising approach for developing advanced therapeutic platforms with broad-spectrum applications in bone regeneration (Table 2).

**Table 2:** Summary of example inorganic materials for bone regeneration

Category	Cement	Active principle	Main results	Refs.
Inorganic ions	CPC	Sr^2+^	Improve compressive strength and prolonging setting time Promote alkaline phosphatase activity and calcium nodule formation Upregulation of VFGF and Ang-1 expression	[86]
	CPC	Cu^2+^	Cu^2+^ ions (0.01–0.74 mg/mL) enhance compressive strength and injectability, while prolonging setting time The optimal concentrations (0.01–0.05 wt% CuP) promote osteogenesis and angiogenesis	[87]
	CPC	Li^+^	Li/CPC has favourable bioactivity in vitro to the same extent as that of CPC Lithium ions enhance biocompatibility and osteogenesis through activation of the Wnt/β-catenin signaling pathway	[89]
	CS/CPC	Zn^2+^/Sr^2+^	Adding two ions to bone cement can produce a synergistic effect Zn/Sr slightly prolongs the setting time of bone cement and has little effect on compressive strength Increase viability, adhesion, proliferation, and expression of osteoblast-related genes in osteoblast-related cells	[90]
	CPC	Si^4+^/Zn^2+^	Improve setting time, injectability and compressive strength Si/Zn dual elements switch macrophage polarity from the M1 phenotype into the M2 phenotype Enhance the osteogenic differentiation of BMSCs	[91]
Bioactive glasses	CPC	Mg^2+^/BG	2 Mg-BG-800-BC displays notably higher Mg ion release (0.41 ± 0.07 mg/L) BG-controlled Mg ion release significantly enhanced BMSCs migration and their secretion of bioactive molecules beneficial for bone regeneration BG-BC surface effectively modulated bone microenvironment via interactions with RAW264.7 and BMSCs	[99]
	CPC	Sr^2+^/MBG	MBG creates pores for tissue ingrowth and releases osteostimulative silicon ions through its controlled degradation Support better vascularization while facilitating the sustained release of strontium ions from the cement matrix	[101]
Other bioceramics	CPC	β-TCP	β-TCP particles (200–450 μm) can shorten the setting time and improve the mechanical strength of bone cement Formation of a tight bond between bone and CPC matrix after hydration	[107]
	CPC	Mn^2+^/β-TCP	Mn^2+^/β-TCP can enhance CPC surface properties (increased protein adsorption capacity and improved cellular response)	[108]
	PMMA	CS	CS can release Ca ions to promote the formation of hydroxyapatite Si ions released by CS can stimulate the proliferation and osteogenic differentiation of MSCs and promote angiogenesis Maintain similar compressive properties to pure PMMA while reducing setting times	[112]
	CPC	ZS/CS	The incorporation of ZS and CS into CPC induces the release of Zn, Ca and Si ions CS/ZS dual compound to CPC considerably enhance the osteogenic differentiation of stem cells	[113]

### Inorganic ions

**Fig. 2:**
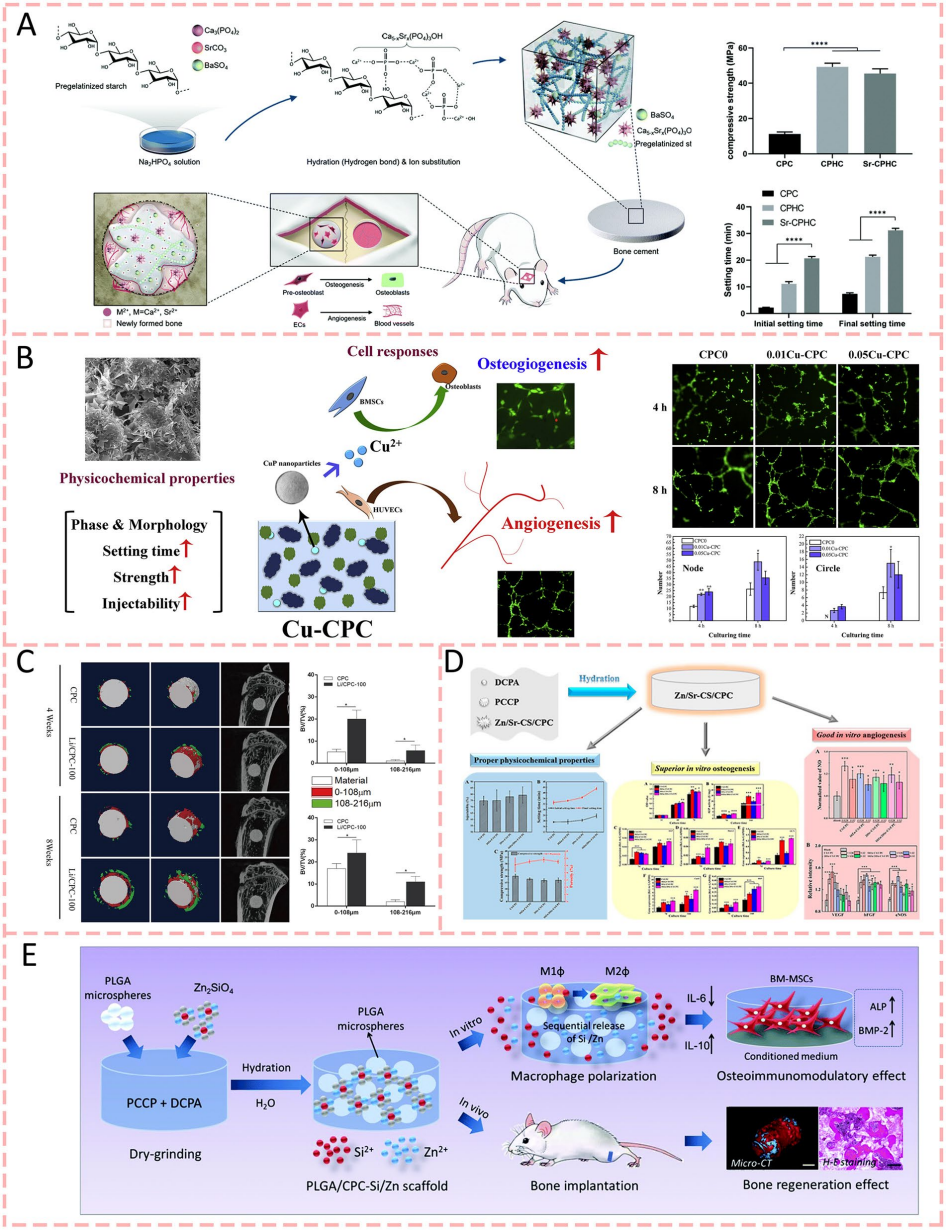
Inorganic ions-modified bone cements for bone regeneration. **A** Schematic diagrams of the strontium ions incorporate into calcium phosphate hybrid cement and their application for bone regeneration engineering [86]. Copyright 2021, Royal Society of Chemistry. **B** A novel copper-doped calcium phosphate cement (Cu-CPC) was reported by incorporating copper phosphate. Controlled release of Cu^2+^ ions enhanced compressive strength, injectability, and improved osteogenesis and angiogenesis [87]. Copyright 2020, Elsevier. **C** Lithium chloride-doped calcium phosphate cement (Li/CPC) demonstrated enhanced biocompatibility and osteogenesis [89]. Copyright 2017, Springer Nature. The images reproduced with the permission from D) The incorporation of Zn and Sr ions into calcium silicate/calcium phosphate cement (CS/CPC) played better and synergistic roles in the viability, adhesion, and proliferation of osteogenesis-related cells [90]. Copyright 2023, American Chemical Society. **E** Si and Zn can promote osteogenesis by improving the bone microenvironment and osteoinductivity synergistically [91]. Copyright 2020, Royal Society of Chemistry

Various inorganic ions, like magnesium (Mg), calcium (Ca), strontium (Sr), lithium (Li), copper (Cu), and zirconium (Zr), exhibit the capacity to enhance bone regeneration through multiple mechanisms, including modification of the bone microenvironment, modulation of immune responses, regulation of cellular activities, and facilitation of both vascular and neural development [85]. When these ions are complexed into bone cement alone or together, they can even change the properties of the cement. In 2021, Wu et al. (2021) [86] found that the incorporation of strontium ions through SrCO3 into calcium phosphate hybrid cement (Sr-CPHC) demonstrated controlled Sr^2+^ release, which significantly enhanced its physicochemical properties by improving compressive strength from 11.21 MPa to 45.52 MPa and prolonging setting time from 2.2 to 20.7 min. Meanwhile, Sr^2+^ greatly improved both osteogenesis by promoting alkaline phosphatase activity and calcium nodule formation in MC3 T3-E1 cells, and angiogenesis by facilitating human umbilical vein endothelial cells (HUVEC) migration and tube formation through upregulation of vascular endothelial growth factor (VEGF) and Ang-1 expression, ultimately leading to improved vascularized bone regeneration as verified in rat calvarial defect models (Fig. 2a). Lin et al. (2020) [87] synthesized a novel copper-doped calcium phosphate cement (Cu-CPC) by incorporating copper phosphate (CuP), which demonstrated that the controlled release of Cu^2+^ ions (0.01–0.74 mg/mL) enhanced compressive strength and injectability while prolonging setting time, and the optimal concentrations (0.01–0.05 wt% CuP) promoted osteogenesis and angiogenesis through improved cell adhesion, proliferation and upregulated expression of bone-related genes in mouse bone marrow stromal cells and human umbilical vein endothelial cells (Fig. 2b). Lithium has been extensively studied and widely applied in battery technology [88]. Beyond its prominent role in energy storage, Li ions have demonstrated remarkable potential in biomedical applications. For example, research [89] has revealed that lithium ions released from lithium chloride-doped calcium phosphate cement (Li/CPC) at concentrations of 25.35–50.74 mg/L demonstrated enhanced biocompatibility and osteogenesis through activation of the Wnt/β-catenin signaling pathway, promoting osteoblast proliferation and differentiation (Fig. 2c). Sometimes, adding two ions to bone cement at the same time can often produce a better synergistic effect. The Zn and Sr played better and synergistic roles in the viability, adhesion, proliferation of osteogenesis-related cells, and the expression of osteogenesis-related genes. The incorporation of Zn and Sr ions into calcium silicate/calcium phosphate cement (CS/CPC) resulted in a slightly prolonged setting time with minimal impact on compressive strength, while demonstrating enhanced biological performance through improved osteogenesis and angiogenesis [90] (Fig. 2d). With PLGA microspheres and Si/Zn dual elements incorporated into CPC scaffolds, setting time, injectability, and compressive strength have been improved. In vivo, Si/Zn dual elements switched macrophage polarity from the M1 phenotype into the M2 phenotype and considerably enhanced the osteogenic differentiation of BMSCs. It is evident from these findings that Si and Zn can promote osteogenesis by improving the bone microenvironment and osteoinductivity synergistically [91] (Fig. 2e). MPC exhibits remarkable osteoinductive properties through sustained Mg^2+^ release. Specifically, Mg^2+^ directly promotes mesenchymal stem cell differentiation into osteoblasts while orchestrating immune responses in the bone microenvironment [92]. In terms of innate immunity, Mg^2+^-based materials facilitate macrophage polarization towards the M2-like phenotype that supports mineralization while suppressing the inflammatory M1-like phenotype [93]. Regarding adaptive immunity, Mg^2+^ supplementation inhibits CD4 + and CD8 + T cell activation, enhances immunosuppressive Treg differentiation, and establishes a microenvironment favorable for cellular differentiation, angiogenesis, and osteogenesis. Substantial evidence from both large animal studies and in vitro/in vivo investigations has demonstrated MPC's efficacy in promoting bone regeneration and repair of critical-sized defects [94]. Kaiser et al. [95] demonstrated that magnesium ions enhance bone regeneration through dual mechanisms, encompassing direct promotion of MSC-to-osteoblast differentiation and immune modulation via M2 macrophage polarization, and T cell response regulation, thereby establishing an optimal microenvironment for osteogenesis, angiogenesis, and mineralization. As a result, it can be beneficial to incorporate multiple trace elements into bone cements to enhance bone regeneration.

### Bioactive glasses

**Fig. 3:**
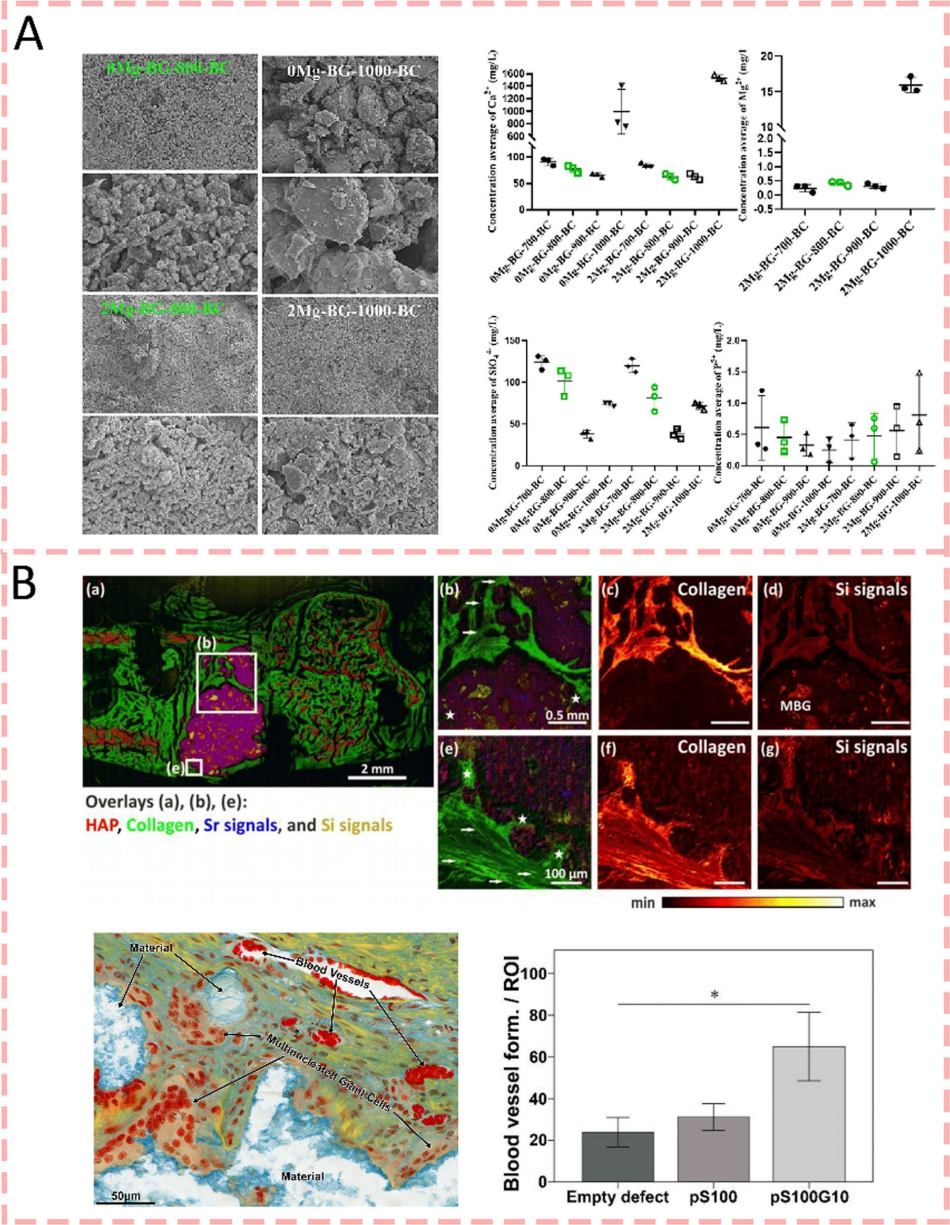
Bioactive glasses-modified bone cements for bone regeneration. **A** The controlled release (Ca, Mg, and Si ions) of ion release of BG-BC compositions effectively modulated the bone microenvironment and was conducive to bone regeneration [99]. Copyright 2024, Elsevier. **B** An injectable calcium phosphate cement doped with strontium and incorporating mesoporous bioactive glass generates a silica gel layer on its surface, which enhances both osteogenic and angiogenic activities [101]. Copyright 2023, MDPI

The bone-bonding, osteogenic, and angiogenic properties of bioactive glasses (BGs) have led to the extensive investigation of BGs as biomedical materials for tissue regeneration [96]. BGs imitate the inorganic components that make up bones through their composition of SiO_2_, Na_2_O, CaO, and P_2_O_5_ [97]. Despite being classified as a bioactive ceramic, BGs are widely used in bone repair applications due to their unique physicochemical properties and outstanding biological performance, warranting a separate review. There are generally three types of BG: traditional BGs, mesoporous bioactive glass (MBG), and ion-doped bioactive glasses (containing Sr, Mg, Zn, and Cu). Recent studies have shown that the biological activity of bioactive glass mainly has two mechanisms, the formation of a bioactive hydroxyapatite layer that facilitates direct bone bonding, and the controlled release of ions that stimulate cellular responses to promote osteogenesis and angiogenesis [85, 98]. In 2024, Dai et al. (2024) [99] demonstrated that Mg-containing bioactive glass modified bone cements exhibited controlled ion release profiles. While 2 Mg-BG-1000-BC showed higher calcium and magnesium ion release (1529.98 ± 50.96 mg/L and 15.93 ± 1.10 mg/L respectively), 2 Mg-BG-800-BC demonstrated more stable and sustained Mg release (0.41 ± 0.07 mg/L). The biological evaluation demonstrated that the BG-BC surface effectively modulated the bone microenvironment through interactions with osteogenic-related cells, RAW246.7, and BMSCs. BMSCs exhibited significantly enhanced migration and secretion of bioactive molecules conducive to bone regeneration, with the 2 Mg-BG-BC group showing a remarkable 12.77-fold increase in cell migration compared to the CaSO4 group, highlighting the crucial role of BG incorporation in creating a favorable microenvironment for bone regeneration through controlled Mg ion release (Fig. 3a). The silica ions released from the BG surface form a silica gel layer on the surface of bone cement, which is followed by the formation of a hydroxyapatite layer due to the amorphous calcium phosphate precipitation. And HAp layer further activates cell migration to trigger bone regeneration [100]. A current work [101] demonstrated enhanced osteogenesis using strontium-doped calcium phosphate cement with mesoporous bioactive glass. ToF–SIMS analysis revealed the spatial distribution of mineralized bone components, including hydroxyapatite layers, collagen, and ion signals (Sr, Si). The interface between cement and tissue showed a porous structure where bioactive glass dissolution and new collagen formation were observed. Furthermore, the fibril structure of fresh collagenous tissue surrounding the bone cement can be visualized within the former defect area. It was also found that CPC-MBG composites can promote angiogenesis around bone cement. (Fig. 3b). Meanwhile, Wekwejt et al. [102] showed that bioactive glass modification improves PMMA bone cement's biological and mechanical properties, with smaller BG particles enhancing distribution and porosity control in 2021. 45S5 BG modification yielded better cytocompatibility and osteogenic capabilities for bone substitutes. There is also a study [103] that shows that surface phytic acid-derived bioactive glass-modified calcium sulfate cement can significantly enhance bone regeneration by promoting sustained hydroxyapatite formation with a PSC content of 55wt%. The PSC/CS cement stimulated robust osteogenesis as evidenced by abundant osteocytes, trabeculae, and vessel formation at the bone-cement interface. Its controlled degradation rate matched new bone formation while maintaining mechanical stability, outperforming both PMMA and CSPC cements for treating vertebral compression fractures. Similar results were demonstrated in the study by Zhang et al. (2022), who developed a pH-neutral phosphosilicate bioactive glass-modified calcium sulfate bone cement that significantly enhanced bone regeneration [104]. Unlike previous studies using conventional alkaline bioactive glasses, this pH-neutral PSC modification avoided interference with cement setting while achieving better bone formation through controlled ion release, particularly silicon ions that stimulated both osteogenesis and angiogenesis.

### Other bioceramics

**Fig. 4:**
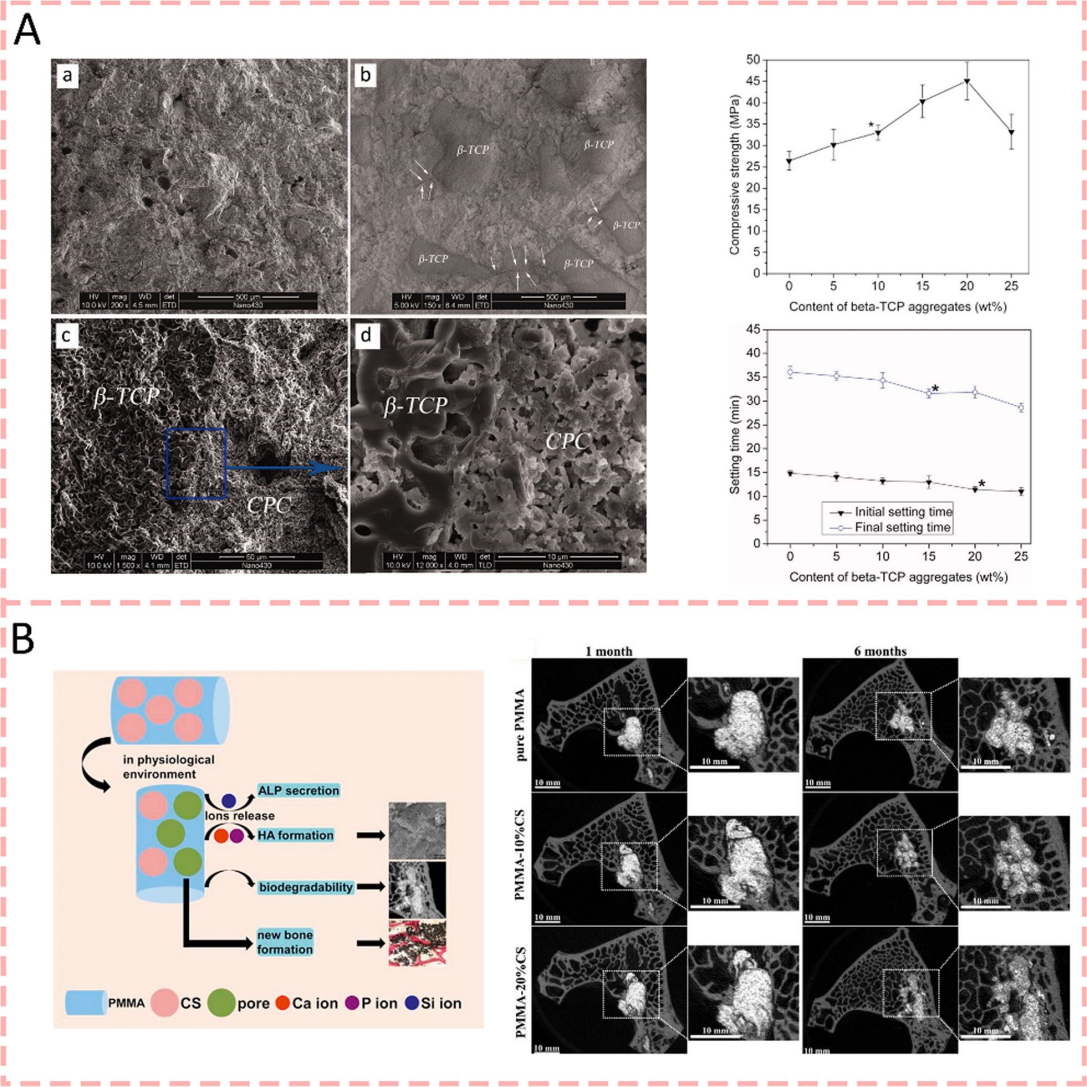
Other bioceramics-modified bone cements for bone regeneration. **A** β-tricalcium phosphate (β-TCP) ceramics added to CPC can shorten the setting time, improve the mechanical strength, and form a tight bond between bone and CPC matrix [107]. Copyright 2012, Wiley Periodicals, Inc. **B** A novel PMMA/CS hybrid cement significantly improved osteogenic capacity by bioactive ion release and hydroxyapatite formation [110]. Copyright 2014, Elsevier

In addition to the above bioactive glasses, there are several uncommon bioceramics that can be compounded with bone cement. β-tricalcium phosphate (β-TCP) ceramics are a type of bioceramics widely used in bone repair with excellent biocompatibility and osteoconductivity [105]. They provide properties for cellular interaction while ensuring excellent biomechanical properties [106]. In addition, a previous study [107] has found that adding β-TCP particles with a particle size of 200 μm to 450 μm to CPC can shorten the setting time of bone cement, improve the mechanical strength, and form a tight bond between bone and CPC matrix after hydration(Fig. 4a). In 2020, Wu et al. (2020) [108] demonstrated that the incorporation of Mn^2+^-doped β-TCP into calcium phosphate cement enhanced its surface properties, leading to increased protein adsorption capacity and significantly improved cellular response, as evidenced by superior adhesion and spreading of mouse bone marrow mesenchymal stem cells on the modified cement containing 20 wt% Mn-TCP. Calcium silicate ceramics are another type of bioceramics, which possess excellent bioactivity, biodegradability, and bone regeneration capability [109]. CS can release Ca ions to promote the formation of hydroxyapatite with high osteoinductive capacity, ultimately promoting the formation of new bone [110]. Moreover, Si ions released by CS can stimulate the proliferation and osteogenic differentiation of mesenchymal stem cells, as well as promote angiogenesis, further accelerating bone regeneration [109, 111]. Sun et al. [112] fabricated novel PMMA/CS hybrid cements that maintained similar compressive properties to pure PMMA while demonstrating reduced setting times. In simulated body fluid, bioactive ion release and hydroxyapatite formation could be detected and osteogenic capacity significantly improved as evidenced by the new bone formation in defect sites after 6 months post-injection (Fig. 4b). The latest research [113] shows that two bioactive ceramic particles, CS and Willemite-phase zinc silicate (Zn2SiO4, ZS), were added to CPC to improve its osteogenic activity by Qian et al. (2024) Zn, Ca, and Si ions were detected after the incorporation of ZS and CS into the CPC. In vitro, results showed that the addition of CS/ZS dual compound to CPC considerably enhanced the osteogenic differentiation of stem cells compared with the incorporation of CS or ZS alone. Moreover, the hybrid cement accelerated the bone regeneration of rabbit femur condyle defects, which is a promising graft strategy for enhancing bone regeneration.

## Applications of organic materials modification strategies for bone cement

### Organic acid-related materials

Organic acid-related materials play a significant role in bone regeneration by influencing mineralization and osteoconductivity. These compounds, such as citrate and malate, are known to facilitate calcium ion release, thereby enhancing the formation of hydroxyapatite. Additionally, organic acid can improve the mechanical properties of bone graft materials and support cellular activities crucial for bone regeneration [114]. Their incorporation into bone cements and scaffolds offers a promising strategy for improving the efficacy of orthopedic and dental applications, ultimately leading to accelerated and more efficient bone regeneration (Table 3).

**Table 3:** Summary of example organic materials for bone regeneration

Category	Cement	Active principle	Main results	Refs.
Organic acid	MPC	Citrate	Achieve initial setting times of 4–8 min and final set times of 15–25 min Exhibit controlled citrate release characteristics and induced moderate alkalinization (pH 7.5–8.5) Effectively promote osteoblast differentiation while simultaneously inhibit osteoclast formation	[68]
	MPC	CaCO3/CA	MPC containing 3% CaCO3/CA exhibited optimal physicochemical properties Superior cell proliferation and adhesion Achieve comparable bone regeneration to commercial Bio-Oss® bone powder	[18]
	CPC	Magnesium malate	MCPC shows appropriate mechanical properties, controlled ion release, and suitable degradation behavior Magnesium malate promotes bone healing via Mg^2+^-PGE2-CGRP signaling pathway through cell–cell interactions	[119]
	Brushite − Collagen Bone Cements	L-tartaric acid	L-tartaric acid selectively binds to the plane of brushite crystals, leading to a 30% increase in fracture toughness Collagen improves cell morphology and increases focal adhesion expression	[120]
Natural polymer	CPC	Collagen	Addition of 5% collagen increase toughness by tenfold compared to pure CPC Maintain flexural strength (8–10 MPa) A two-fold increase in osteoblast cell attachment with improved cell viability and spreading	[129, 130]
	PMMA	Mineralized collagen	Reduce compressive strength and match natural bone tissue Improve BMSC adhesion, proliferation, and osteogenic differentiation	[131]
	CMPC	Alginate sodium	AS coating enables controlled and sustained release of Mg^2+^ and Ca^2+^ ions Promote enhanced osteogenesis through the TRPM7/PI3 K/Akt signaling pathway	[41]
	MPC	CMCS/SA	Enhance the mechanical properties and handling characteristics of MPC Create an optimal microenvironment for bone regeneration through the Wnt/β-catenin signaling pathway	[79]
	MPC	Carboxymethyl chitosan	5% CMC shows optimal compressive strength Promote MC3 T3-E1 adhesion, proliferation, and differentiation through fibronectin adsorption and activated integrin signaling through increased FAK and ERK phosphorylation	[67]
	PMMA	Hyaluronic acid	HA enhances cell attachment by providing binding sites for osteoblasts and osteoprogenitor cells HA binds and regulates growth factors like BMPs to enhance osteogenic differentiation Improve surface properties to promote protein adsorption and cell-material interactions	[136]
Synthetic polymer	CPC	PLGA	Effectively enhance CPC degradation through controlled acid release Maintain biocompatibility	[142]
	CPC	PCL	Enhance degradation rate and bone regeneration through the creation of macroporosity	[143]
	CPC	PVA	Serve as an injectable composite matrix to improve mechanical properties	[144]

**Fig. 5:**
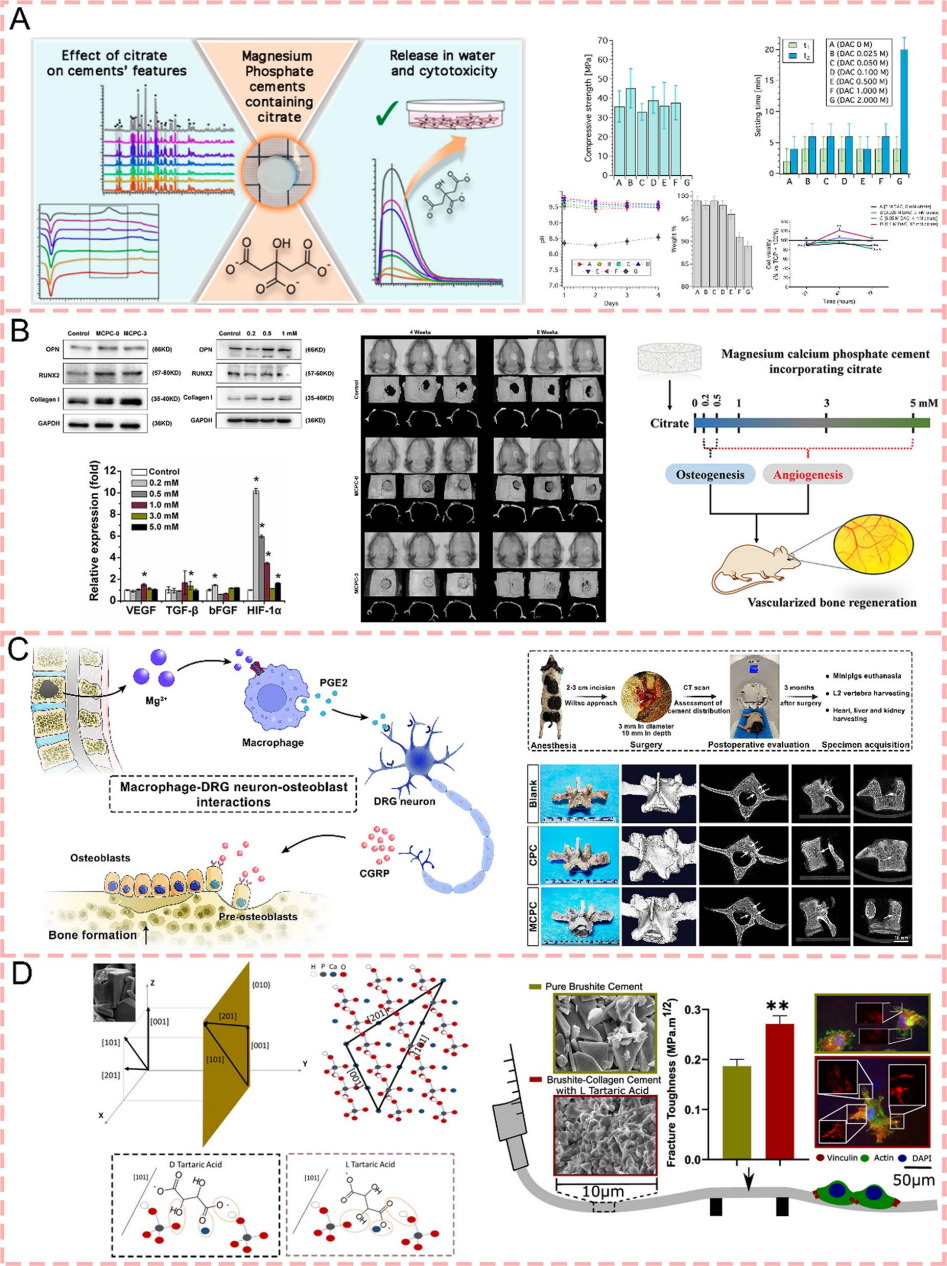
Organic acid-modified bone cements for bone regeneration. **A** Schematic of the citrate-modified MPCs, the incorporation of moderate citrate concentrations maintained their compressive strength and induced mild alkaline conditions, which effectively promoted osteoblast differentiation while inhibiting osteoclast formation [68]. Copyright 2020, American Chemical Society. **B** A novel MPC formulation by incorporating citric acid as a modifier into a composite system enhances vascularized bone regeneration [117]. Copyright 2019, Elsevier. **C** Schematic diagrams of the magnesium malate-modified CPC (MCPC) and their application for bone regeneration engineering. They can facilitate interactions among macrophages, sensory neurons, and osteoblasts [119]. Copyright 2024, Springer Nature. **D** L-tartaric acid added to cement can selectively bind to the plane of brushite crystals, improve cell morphology, and increase focal adhesion expression [120]. Copyright 2020, American Chemical Society

Citrate, a naturally occurring organic component in human bone tissue, accounts for approximately 90 wt% accumulation in the skeletal system [115]. As a crucial mineralization regulator, it controls the size and crystallinity of mineral hydroxyapatite platelets [116]. This intrinsic role of citrate in bone physiology establishes it as a vital bridge between cellular mechanisms and biomaterial innovations. Gelli et al. [68] loaded citrate on MPCs as a therapeutic agent. They used Seven solutions with different contents of diammonium hydrogen phosphate (DAHP, (NH4)2HPO4, purity ≥ 99%) and dibasic ammonium citrate monohydrate (DAC, OCCOOH (CH2 COONH4)2·H2O, purity ≥ 99%) into MPCs, where citrate is used to modify viscosity. The experimental results revealed that when citrate was introduced at optimal concentrations (DAC/DAHP molar ratio of 0.014–0.029), it served as an effective setting modifier, achieving initial setting times of 4–8 min and final setting times of 15–25 min, making these formulations suitable for surgical applications. The incorporation of moderate citrate concentrations demonstrated no adverse effects on the compressive strength of the cements. Upon exposure to aqueous environments, these cement formulations exhibited controlled citrate release characteristics and induced moderate alkalinization (pH 7.5–8.5), with degradation rates manifesting as 2–5% weight loss over four days. Most significantly, at optimal concentrations (2–12 mM released citrate), the citrate-modified MPCs demonstrated excellent biocompatibility, with the highest performing formulation (12 mM citrate) showing approximately 115–120% cell viability compared to TCP control at 48 h (Fig. 5a), suggesting superior cytocompatibility compared to traditional calcium phosphate cements. Wang et al. [117] also developed a novel MPC formulation by incorporating citric acid as a modifier into a composite system consisting of MgO, KH2P2O4, and Ca(H2PO4)2 particles. This citric acid-modified MPC demonstrated exceptional properties, with the optimal formulation (2wt% citric acid, MCPC-2) exhibiting extended setting time from 11 to 14 min, enhanced compressive strength up to 76 MPa (compared to control MCPC), reduced porosity while maintaining excellent cytocompatibility and notable osteoinductive capacity. The comprehensive evaluation of these characteristics suggests that this innovative cement formulation holds considerable promise as a biomaterial for bone regeneration applications. Wu et al. [118] investigated the functional effects of citrate-incorporated MPC on bone regeneration. Their research demonstrated that citrate exhibits optimal osteogenic effects at low concentrations (around 200 μM) by enhancing osteogenic markers expression and mineral formation, while promoting angiogenesis through upregulation of angiogenic factors. The study revealed that citrate not only improves MPC's physical properties but also regulates cellular energy metabolism through SLC13a5 transporters and influences key vascular proteins (PPAR-γ and Edf1). Using both in vitro cell studies and a rat skull defect model, they established that citrate-modified MCPC provides a promising strategy for vascularized bone regeneration, though noting that high concentrations (5.0 mM) may inhibit cellular ATP synthesis and proliferation. This work significantly advances our understanding of how citrate concentration can be optimized in bone cement materials to achieve both osteogenic and angiogenic effects simultaneously. This citric acid-modified bone cement is also used in dental diseases. Wang et al. [18] developed a porous magnesium phosphate cement (MPC) by incorporating calcium carbonate (CaCO3) and citric acid (CA) as foaming agents. Their research showed that MPC containing 3% CaCO3/CA exhibited optimal physicochemical properties, including enhanced porosity, improved injectability, extended setting time, and reduced hydration temperature. The modified MPC demonstrated superior cell proliferation and adhesion compared to conventional MPC. In vivo studies using rat periodontal bone defect models revealed that the 3% CaCO3/CA-MPC achieved comparable bone regeneration to commercial Bio-Oss^®^ bone powder. These improvements in both physical properties and biological performance suggest that this porous MPC system offers promise as a bone regeneration material (Fig. 5b).

A recent study explores [119] the novel application of magnesium malate, a clinically established magnesium supplement, in CPC modification. This magnesium malate-modified CPC (MCPC) demonstrates unique advantages in preventing cement collapse in aqueous environments. MCPC achieves a compressive strength of 6.18 ± 0.49 MPa, with a controlled magnesium ion release profile reaching 318.49 ± 3.59 mg/L over 35 days. The material shows gradual degradation with a cumulative rate of 17.26 ± 0.12% at 35 days and an optimal setting time of 38.7 ± 1.7 min. These properties, combined with its straightforward preparation process, make it particularly suitable for clinical applications in blood-rich environments. Notably, the study reveals that magnesium malate enhances bone regeneration through the Mg^2+^-PGE2-CGRP axis, facilitating interactions among macrophages, sensory neurons, and osteoblasts. The osteogenic potential of MCPC was successfully validated through in vivo studies using a minipig vertebral model, establishing its potential as a promising bone cement material (Fig. 5c). Sarrigiannidis et al. (2020) [120] developed an enhanced brushite-collagen type I cement utilizing chiral chemistry, specifically employing L-tartaric acid which selectively binds to the plane of brushite crystals. This selective binding results in controlled crystal growth and improved crystal packing, leading to a 30% increase in fracture toughness compared to conventional brushite cement. The incorporation of collagen further enhanced the biological properties, demonstrating improved cell morphology and increased focal adhesion expression. This innovative approach combining chiral chemistry with biomaterials engineering produced a mechanically superior and biologically enhanced bone cement system (Fig. 5d).

### Natural or synthetic polymer-related materials

Polymeric materials have gained significant prominence in medical applications owing to their superior biocompatibility and biodegradable properties [121]. When incorporated into bone cements, these materials enhance biological performance by introducing multifunctional characteristics. The modified composites serve as temporary scaffolds that provide structural support and fill tissue defects while facilitating controlled degradation. This temporal support enables gradual bone regeneration as the material is systematically resorbed and replaced by newly formed bone tissue [122]. Based on their sources, these polymeric materials can be categorized into two main classes: those derived from natural origins and those synthesized artificially [123]. Each category offers unique advantages and specific modifications to cement properties, contributing to their diverse applications in bone tissue engineering (Table 3).

#### Natural polymer

**Fig. 6:**
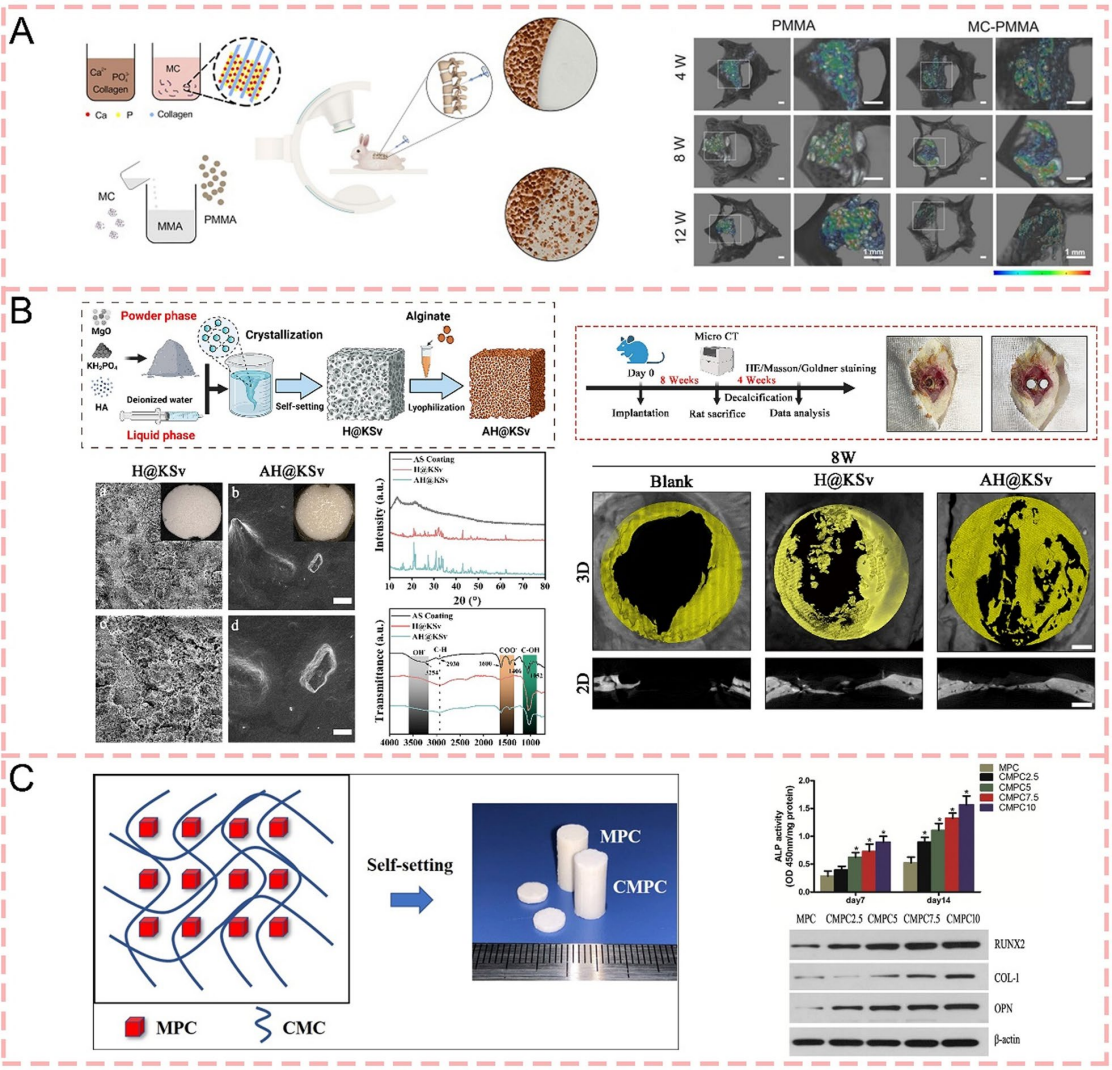
Natural polymer-modified bone cements for bone regeneration. **A** Schematic diagrams of the mineralized collagen (MC)-modified PMMA. They can better match natural bone tissue and demonstrate superior bone regeneration capacity [131]. Copyright 2020, Ivyspring International Publisher. **B** Surface functionalization of calcium-magnesium phosphate cements (CMPCs) with alginate sodium (AS) coating through lyophilization significantly enhanced their performance in bone regeneration [41]. Copyright 2024, Elsevier. **C** Schematic of the chitosan-modified MPC. CMC-MPC promoted MC3 T3-E1 adhesion, proliferation, and differentiation by enhancing fibronectin adsorption and activating integrin signaling through increased FAK and ERK phosphorylation [67]. Copyright 2020, Elsevier

Natural polymers represent a significant class of biomaterials that have attracted considerable attention in medical applications which extracted directly from nature. These materials consist of proteins. Among them, collagen has emerged as the predominant matrix choice for bone regeneration applications [124], while other notable natural polymers include chitosan, alginate sodium, hyaluronic acid (HA), and cellulose [125–127]. The inherent advantages of natural polymers lie in their exceptional biological properties, including biocompatibility, biodegradability, and minimal toxicity [128]. Additionally, their abundant availability and cost-effectiveness make them particularly attractive for industrial applications [128]. These characteristics have established natural polymers as excellent candidates for modification purposes, particularly in bone cement-based systems designed for bone tissue engineering. Their integration with bone cement shows great potential in enhancing the biological performance and functional properties of the resulting composites. Researches [129, 130] show the incorporation of collagen into CPC led to significant improvements in both mechanical and biological properties. The addition of collagen (particularly at 5% concentration) resulted in a tenfold increase in work-of-fracture (toughness), indicating enhanced fracture resistance while maintaining clinically acceptable flexural strength (8–10 MPa). Biologically, the collagen-modified CPC demonstrated a two-fold increase in osteoblast cell attachment, with improved cell viability and spreading. The collagen fibers were effectively integrated into the CPC matrix and became covered with nano-apatite crystals, creating an intimate composite structure that mimics natural bone composition. Zhu et al. (2020) [131] demonstrated that the incorporation of 15% mineralized collagen (MC)-a biomimetic composite of type-I collagen and nano-hydroxyapatite—into PMMA bone cement significantly improved its biological performance while maintaining suitable handling properties. The MC-PMMA composite exhibited reduced compressive strength and modulus that better matched natural bone tissue. In vitro studies revealed enhanced biocompatibility with improved bone mesenchymal stem cell adhesion, proliferation, and osteogenic differentiation. Most notably, the composite demonstrated superior bone regeneration capacity in vivo, as evidenced by increased cortical bone thickness, osteoblast area, and new bone formation in both animal models and clinical studies. These comprehensive improvements suggest that MC-PMMA presents a promising alternative to conventional PMMA cement for treating osteoporotic vertebral compression fractures (Fig. 6a). Another study [132] demonstrated that MC-modified PMMA bone cement offers notable improvements over conventional PMMA cement for treating Kümmell disease. The modification reduced cement leakage rates (10% vs 23.08%) and significantly decreased adjacent vertebral re-fracture incidence compared to unmodified PMMA. While both materials showed similar initial clinical outcomes in terms of Visual Analog Scale (VAS) and Oswestry Disability Index (ODI) scores, the MC-modified PMMA exhibited superior long-term performance at 1-year follow-up. The MC modification appears to address key limitations of conventional PMMA, including its excessive modulus and poor biocompatibility. Though the paper does not explicitly detail cellular osteogenic effects or bone regeneration mechanisms, the improved CT values at 1-year follow-up in the MC-modified group suggest enhanced bone integration and remodeling capabilities.

Alginate sodium serves as a superior surface modifier for bone cements by providing enhanced mechanical properties, controlled ion release, and improved hydrophilicity through simple lyophilization processing, whereas collagen, though bioactive, is typically more expensive, requires complex processing, and lacks the same ion-chelating capabilities. Gong et al. (2024) [41] showed that surface functionalization of calcium-magnesium phosphate cements (CMPCs) with alginate sodium (AS) coating through lyophilization significantly enhanced their performance in bone regeneration. The AS coating improved the mechanical properties and hydrophilicity of the composite scaffolds (AH@KSv), leading to better cell adhesion. More importantly, the AS coating enabled controlled and sustained release of Mg^2+^ and Ca^2+^, which promoted enhanced osteogenesis both in vitro and in vivo through the TRPM7/PI3 K/Akt signaling pathway. The in vitro studies showed increased osteogenic differentiation of MC3 T3-E1 cells, while in vivo experiments revealed improved patterns of new bone formal. These findings provide valuable insights into the mechanism of Mg-based biomaterials in bone regeneration and suggest a promising approach for enhancing the therapeutic efficacy of CMPCs in orthopedic applications (Fig. 6b). Building upon the previous discussion of alginate sodium's capabilities, a study [79] further demonstrates its synergistic potential when combined with carboxymethyl chitosan (CMCS) to modify magnesium phosphate cement. The CMCS/SA gel network not only enhanced the mechanical properties and handling characteristics of MPC but also created an optimal microenvironment for bone regeneration through the Wnt/β-catenin signaling pathway. This composite system demonstrated superior performance in both in vitro osteogenic differentiation and in vivo bone regeneration compared to unmodified MPC, further validating alginate sodium's versatility as a functional modifier in bone cement applications through different molecular mechanisms and combinations.

Chitosan is a natural cationic polymer widely used in biomedicine, while its derivative carboxymethyl chitosan exhibits enhanced biocompatibility, biodegradability, and osteogenic properties [133, 134]. Yu et al. [67] developed a novel injectable bioactive magnesium phosphate cement by incorporating CMC into magnesium phosphate cement for bone regeneration. The addition of CMC significantly improved the physicochemical properties of MPC, including prolonged setting time, reduced setting temperature, enhanced injectability, and improved washout resistance, with 5% CMC showing optimal compressive strength. In vitro studies demonstrated that CMC-MPC specimens promoted MC3 T3-E1 adhesion, proliferation, and differentiation compared to unmodified MPC, which was attributed to enhanced fibronectin adsorption and activated integrin signaling through increased FAK and ERK phosphorylation (Fig. 6c). These findings suggest that the incorporation of CMC into MPC significantly enhanced both its physicochemical properties and osteogenic capacity, demonstrating great potential as an advanced bone regeneration material.

Hyaluronic acid, a naturally abundant polymer, serves as an effective modification material for bone cements [135]. While HA alone shows limited modification capability, its derivatives and composites demonstrate enhanced performance. For instance, hyaluronic acid-polyethylene glycol incorporation into PMMA cement improved both mechanical properties and bone integration [136]. Similarly, curcumin-modified HA combined with glucose microparticles in calcium phosphate cement enhanced porosity, controlled drug release, and accelerated bone regeneration through improved vascularization and osteopontin expression [137]. These applications demonstrate HA's versatility as a modification material for enhancing bone cement performance and osteogenic properties.

#### Synthetic polymer

Synthetic polymers enhance bone cements'injectability and anti-washout properties, though biopolymers often compromise mechanical strength [138]. Poly (lactide-co-glycolic acid) (PLGA) has emerged as the leading synthetic polymer for bone cement modification owing to several unique advantages. Its FDA approval, degradation rate controllability through monomer ratio adjustment, and biocompatible degradation products make it distinctly superior to other synthetic polymers [139]. Its degradation byproducts—lactic acid and glycolic acid—not only facilitate natural metabolism but also create a mildly acidic microenvironment that promotes both cement degradation and vascularization, thereby enhancing bone regeneration [140]. These characteristics make PLGA an exceptional polymer for bone cement modification. The evolution of PLGA-modified CPC systems demonstrates progressive improvements in bone regeneration applications. An initial study [141] in 2011 established that PLGA microspheres could effectively enhance CPC degradation through controlled acid release while maintaining biocompatibility. Maenz et al. [142] advanced this concept by utilizing PLGA fibers, which significantly improved mechanical properties and osteoconductivity in load-bearing applications. The PEGylated poly (glycerol sebacate) modification of CPC demonstrates the versatility of synthetic polymers in bone cement enhancement. The double-crosslinked network strengthens mechanical properties while introducing adhesive capabilities through surface chemical bonding. This organic–inorganic hybrid system effectively combines material improvement with drug delivery function, transforming traditional bone cement into a therapeutic platform for osteoporotic bone regeneration [43]. Poly-ε-caprolactone (PCL) and poly(vinyl alcohol) (PVA) have also been explored as modifying agents for CPC in bone repair applications, where PCL ultrafine fibers can enhance degradation rate and bone regeneration through the creation of macroporosity, while PVA serves as an injectable composite matrix to improve mechanical properties. However, their limitations include the acidic degradation products from PCL that may affect local pH and tissue response and the non-degradable nature of PVA which could potentially interfere with complete bone regeneration and remodeling [143, 144].

## Applications of nanomaterials modification strategies for bone cement

Nanomaterials have emerged as promising modifiers for cementitious composites, demonstrating significant enhancement in mechanical properties, microstructural characteristics, and material durability [145, 146]. These materials can be fundamentally categorized into two main classes: inorganic and organic nanomaterials, each offering distinct advantages in bone cement modification. Inorganic nanomaterials contribute primarily to the mechanical and physiochemical aspects of bone cement. They significantly enhance mechanical strength and structural rigidity while promoting biological activity. Through their ability to facilitate mineralization and release beneficial inorganic ions, these materials effectively simulate the natural bone microenvironment, ultimately supporting successful osseointegration. In contrast, Organic nanomaterials exhibit remarkable capabilities in enhancing the structural and biological properties of bone cement. They effectively improve material toughness and injectability while simultaneously providing essential biological functions. These materials excel in regulating cellular responses and mimicking the extracellular matrix, creating an optimal bone regeneration environment (Table 4).

**Table 4:** Summary of example nanomaterials for bone regeneration

Category	Cement	Active principle	Main results	Refs.
Metal-based nanoparticles	α-TCP/CS biphasic systems	Fe3O4/GO nanocomposites	A 10 wt% Fe3O4/GO formulation exhibits optimal magnetothermal performance Effectively eliminate residual tumor cells while maintaining the cement's structural integrity Preserve essential properties including biocompatibility and osteogenic potential	[148]
	CPC	Iron oxide nanoparticles	Enhance the mechanical strength of the CPC scaffolds Promote the adhesion and spreading of human dental pulp stem cells 3% IONP enhances alkaline phosphatase activity and bone formation by 1.5–2 folds	[149]
	PMMA	Nano-MgO particles	Enhance handling properties when nano-MgO content was below 15 wt% Promote higher calcium nodule formation and increased osteogenic gene expression Superior bone-bonding strength after 12 weeks of implantation	[150]
	CPC	Gadolinium oxide nanoparticles	Enhance the mechanical properties of the bone cement (compressive strength and E-modulus) Promote human osteoblast proliferation	[151]
	CPC	Copper nanoparticle	1 wt% Cu enhance osteogenic potential through increase ALP activity and cell growth Provid additional benefits of angiogenic and antibacterial properties	[152]
Mesoporous silica nanoparticles	MCPC	HMSNs-PTH-ALN	Enhance ion/drug release, controlled degradation, and create a porous structure for cell growth Improve the osteoporotic pathophysiological microenvironment Enhance vascularized bone defect regeneration	[156]
	MPC	MSN-ALN	Significantly enhance the performance of cement (compressive strength, extended setting time, and superior injectability) Improve extracellular matrix mineralization Upregulation of osteogenic genes	[69]
Bioactive glass nanoparticles	CPC	BGn	Enhance surface area, superior protein adsorption capacity, and controlled release of bioactive ions (Si and Ca) Promote osteogenic differentiation of mesenchymal stem cells and angiogenic behavior of endothelial cells Superior osteoinductive and osteoconductive properties	[81]
	α-TCP	Mesoporous bioactive glass nanoparticles	The nanocomposite improved surface area (18.6 m^2^/g), ion release, and protein binding (252 mg/g) Readily form hydroxyapatite layers in vitro	[158]
	Nanoscale cement	Sr-BGn	Promote osteogenesis through enhanced expression of osteogenic genes and proteins Inhibit osteoclastogenesis by reducing osteoclastic activity and bone resorption	[159]
Other Inorganic nanomaterials	CPC	GO-Cu nanocomposite	Graphene oxide serves as a carrier for Cu ions Enhance cell adhesion while enabling uniform scaffold coating Improve the adhesion and proliferation of BMSC	[162]
	PMMA	Amine-functionalized graphene	Demonstrate exceptional osteointegration capabilities while maintaining minimal cytotoxicity Promote calcification in vivo Reduce cellular oxidative stress	[163]
	PMMA	Hydrophilic graphene oxide	Enhance bone-material contact and superior osteogenic capabilities	[164]
	PMMA	Layered double hydroxides	Decrease the maximum polymerization temperature by 7.0 °C Alleviate stress-shielding osteolysis and indirectly promote osseointegration Release of magnesium ions and create a favorable microenvironment	[167]
Polymer nanomaterials	CPC	PLGA nanofibers	Reduce CPC brittleness and improve its mechanical properties PLGA nanofibers create pores during degradation Release bioactive factors and create an acidic environment	[168]
	CPC	Short PLGA nanofibers	Dynamically controllable biodegradability PLGA-released lactic acid enhances vascularization	[169]
	MPC	Electrospun silk fibroin	The hierarchical structure enables rapid oxygen and nutrient infiltration Improve MPC strength and neutralize its alkaline environment	[42]

### Inorganic nanomaterials

#### Metal-based nanoparticles

**Fig. 7:**
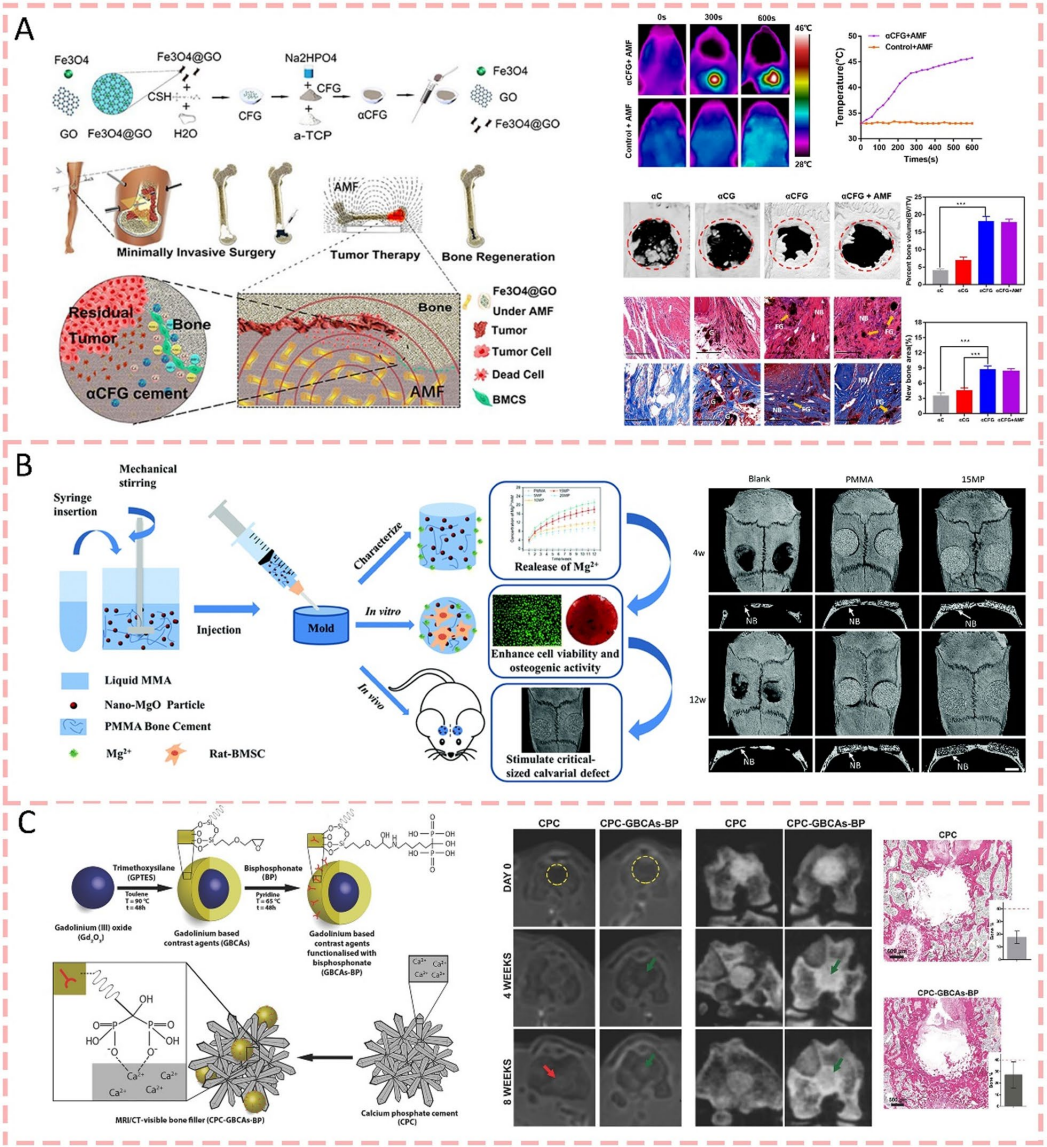
Metal-based nanoparticles modified bone cements for bone regeneration. **A** Schematic diagram of Fe3O4/GO nanocomposites-modified bone cement, which achieves dual functions of magnetic hyperthermia and osteogenesis [148]. Copyright 2019, American Chemical Society. **B** Incorporating nano-MgO particles into PMMA bone cement resulted in enhanced new bone formation, handling properties, and bone-bonding strength [150]. Copyright 2020, Royal Society of Chemistry. **C** Overview schematic of gadolinium oxide nanoparticles was employed to modify CPC. The functionalized nanoparticles enabled long-term MRI/CT multimodal imaging and promoted human osteoblast proliferation [151]. Copyright 2019, WILEY–VCH Verlag GmbH

Metal-based nanoparticles (MNPs), particularly metal oxide nanoparticles, have gained significant attention in biomedical sciences and engineering [147]. Fe3O4/GO (iron oxide/graphene oxide) nanocomposites were incorporated into α-TCP/CS biphasic systems to develop an innovative injectable bone cement for treating tumor-induced bone defects. Through optimization studies, a 10 wt% Fe3O4/GO formulation exhibits optimal magnetothermal performance. Under alternating magnetic field stimulation, this system achieves controlled heating between 43 and 46 °C, which effectively eliminates residual tumor cells while maintaining the cement's structural integrity. Beyond its therapeutic functionality through magnetic hyperthermia, the Fe3O4/GO modification preserves essential properties including biocompatibility and osteogenic potential. This dual-functional bone cement system thus presents a promising minimally invasive approach for concurrent bone reconstruction and tumor treatment in clinical settings (Fig. 7a) [148]. Similarly, Xia et al. (2019) [149] incorporated iron oxide nanoparticles (IONP) into CPC as a powder form to prepare composite scaffolds. The addition of IONP significantly enhanced the mechanical strength of the CPC scaffolds. In terms of biological performance, the IONP-modified cement substantially promoted the adhesion and spreading of human dental pulp stem cells (hDPSCs). Notably, with 3% IONP incorporation, the alkaline phosphatase activity, osteogenic gene expression, and bone matrix formation were increased by 1.5–2 folds. While iron oxides have been explored as bone cement modifiers, magnesium oxide (MgO) presents unique advantages due to its essential role in bone metabolism and its ability to release Mg^2+^ ions that directly influence osteogenic activity. A study demonstrated that incorporating nano-MgO particles into PMMA bone cement resulted in several significant improvements. The modified cement showed enhanced handling properties when nano-MgO content was below 15 wt%. Although the compression modulus and strength decreased with MgO addition, the modified cement exhibited superior biocompatibility compared to pure PMMA cement. In vitro studies revealed that nano-MgO/PMMA promoted higher calcium nodule formation and increased osteogenic gene expression in rat BMSCs. Furthermore, in vivo experiments using a rat calvarial defect model demonstrated that nano-MgO/PMMA significantly enhanced new bone formation, achieving 50% greater bone mineral density compared to unmodified PMMA. The modified cement also demonstrated superior bone-bonding strength after 12 weeks of implantation (Fig. 7b) [150]. These findings suggest that nano-MgO/PMMA bone cement holds promising potential for joint fixation and bone defect-filling applications. Gadolinium oxide nanoparticles were employed to modify CPC in a novel study conducted by Mastrogiacomo et al. (2018) [151]. The modified nanoparticles were functionalized with bisphosphonate groups, which significantly improved their binding affinity to the CPC matrix. This modification resulted in several beneficial changes. First, it enhanced the mechanical properties of the bone cement, including increased compressive strength and E-modulus. Second, the functionalized nanoparticles enabled long-term MRI/CT multimodal imaging of the implanted cement, addressing the challenge of monitoring CPC performance in vivo. Additionally, in vitro studies demonstrated that at lower concentrations, the modified nanoparticles promoted human osteoblast proliferation, suggesting potential benefits for bone regeneration (Fig. 7c) [151]. Meanwhile, Pillai et al. (2023) [152] pointed out that copper nanoparticle-modified CPC scaffolds, particularly at 1 wt% Cu loading, showed improved osteogenic potential through enhanced alkaline phosphatase activity and better cell proliferation compared to unmodified CPC, while also providing additional benefits of angiogenic and antibacterial properties.

#### Mesoporous silica nanoparticles

**Fig. 8:**
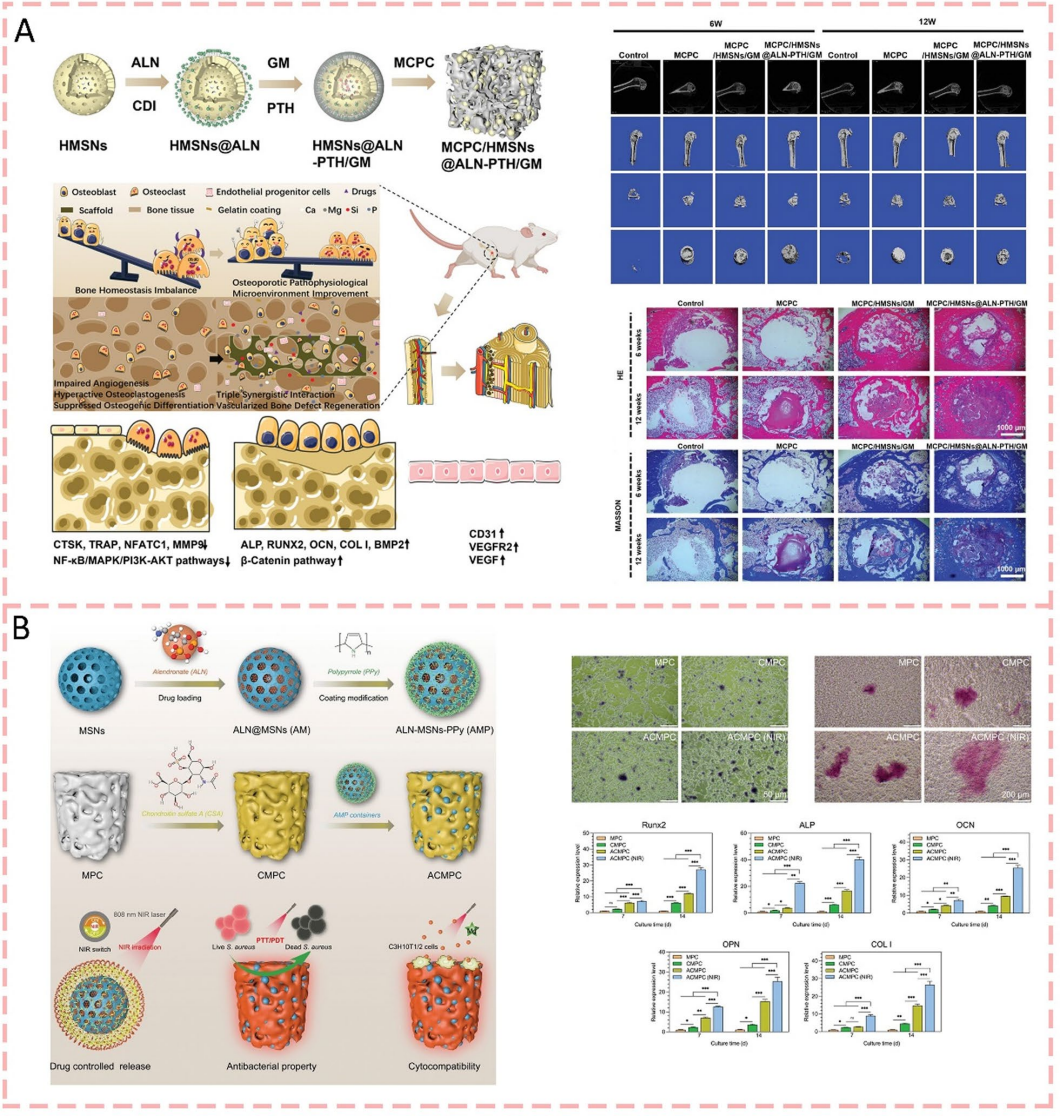
Mesoporous silica nanoparticles modified bone cements for bone regeneration. **A** Schematic showing a multifunctional scaffold where PTH and ALN-loaded HMSNs/GM were incorporated into calcium magnesium phosphate cement to enhance vascularized bone regeneration in osteoporotic conditions [156]. Copyright 2023, WILEY–VCH Verlag GmbH. **B** Schematic of the modified MPC system with MSNs loaded with sodium alendronate, which showed excellent biocompatibility and enhanced osteogenic potential [69]. Copyright 2024, WILEY–VCH Verlag GmbH

As early as 2014, mesoporous silica nanoparticles were studied as reinforcing agents in PMMA bone cement. The addition of MSNs improved the flexural modulus and compressive properties but decreased flexural strength, fracture toughness, and fatigue resistance due to poor interfacial adhesion between MSNs and the polymer matrix [153, 154]. In 2017, MSNs were successfully employed as drug delivery platforms in PMMA bone cement. When loaded with gentamicin, MSN-modified bone cements (8.15 wt%) maintained their mechanical properties for up to 6 months while achieving enhanced antibiotic release compared to commercial antibiotic-loaded bone cements [155]. Recently, Zhao et al. [156] developed a multifunctional scaffold by incorporating gelatin-coated hollow mesoporous silica nanoparticles (HMSNs/GM) loaded with parathyroid hormone (PTH) and alendronate (ALN) into calcium magnesium phosphate cement. This modification significantly enhanced the properties of the bone cement in several aspects, such as spatial–temporal release of therapeutic ions and drugs, a controllable degradation rate, and an abundant porous structure beneficial for cell growth and vascularization. When tested in ovariectomized rats, this multifunctional scaffold effectively improved the osteoporotic pathophysiological microenvironment and enhanced vascularized bone defect regeneration (Fig. 8a). The performance of cement was significantly enhanced by the modified MPC system with MSNs loaded with sodium alendronate, including improved compressive strength (70.6 ± 5.9 MPa), extended setting time (913 s), and superior injectability (96.5% injection rate). Most notably, the system showed excellent biocompatibility and enhanced osteogenic potential, as evidenced by increased alkaline phosphatase activity, improved extracellular matrix mineralization, and upregulation of osteogenic genes. These improvements make this modified cement system particularly promising for clinical bone repair applications (Fig. 8b) [69]. These observations are consistent with Wang et al.’s (2024) [157] previous investigations.

#### Bioactive glass nanoparticles

**Fig. 9:**
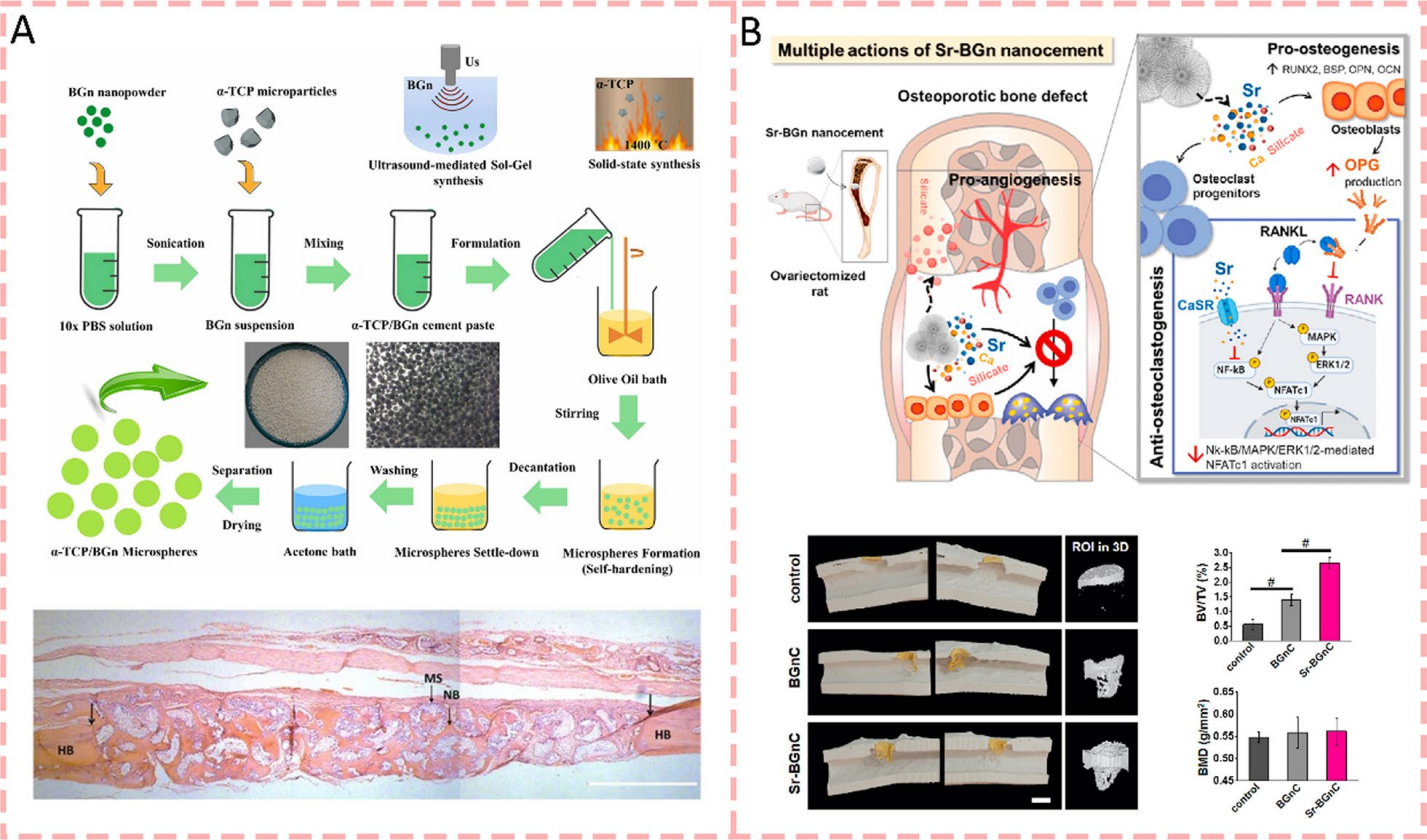
Bioactive glass nanoparticles modified bone cements for bone regeneration. **A** Schematic of the engineered bone cement microspheres modified by mesoporous bioactive glass nanoparticles, which demonstrated controlled silicate ion release, superior protein binding capacity, and exceptional bioactive potential [158]. Copyright 2022, Elsevier. **B** Schematic illustrating strontium-incorporated bioactive glass nanoparticles in cement system, which enables sustained multi-ion release to enhance osteogenesis while inhibiting bone resorption [159]. Copyright 2021, Elsevier

Mesoporous bioactive glass nanoparticles (BGn) are characterized by their unique mesoporous structure, excellent bioactivity, and controlled ion release properties. Kang et al. [81] synthesized a novel nanocement by utilizing BGn as the primary component. This innovative approach significantly improved the cement's properties compared to conventional CPC. The nanocement exhibited enhanced surface area, superior protein adsorption capacity, and controlled release of bioactive ions (Si and Ca). Furthermore, the released ions effectively stimulated cellular responses, promoting osteogenic differentiation of mesenchymal stem cells and angiogenic behavior of endothelial cells. Most notably, in vivo studies demonstrated that the nanocement possessed superior osteoinductive and osteoconductive properties, suggesting its promising potential for bone tissue regeneration applications. Similarly, researchers engineered innovative bone cement microspheres by combining mesoporous bioactive glass nanoparticles with α-tricalcium phosphate (α-TCP). This nanocomposite design yielded remarkable improvements in the cement's fundamental properties, including enhanced surface area (18.6 m^2^/g), controlled silicate ion release, and superior protein binding capacity (252 mg/g). The microspheres demonstrated exceptional bioactive potential by readily forming hydroxyapatite layers in vitro. When implanted in rat skull defects, these microspheres facilitated substantial bone regeneration within 6 weeks. By adopting a microsphere architecture rather than conventional solid cement blocks, the material achieved better biological interaction and created optimal spaces for new bone growth (Fig. 9a) [158]. The strontium-releasing nanoscale cement (Sr-nanocement) was synthesized by incorporating strontium ions into bioactive glass nanoparticles, which maintained desirable cement properties including self-hardening ability and mesoporous structure. What distinguishes this work is its demonstration of dual therapeutic actions. The Sr-nanocement not only promoted osteogenesis through enhanced expression of osteogenic genes and proteins but also inhibited osteoclastogenesis by reducing osteoclastic activity and bone resorption. The sustained release of multiple ions (Si, Ca, and Sr) at therapeutically relevant doses played a key role in these effects (Fig. 9b) [159].

#### Others

**Fig. 10:**
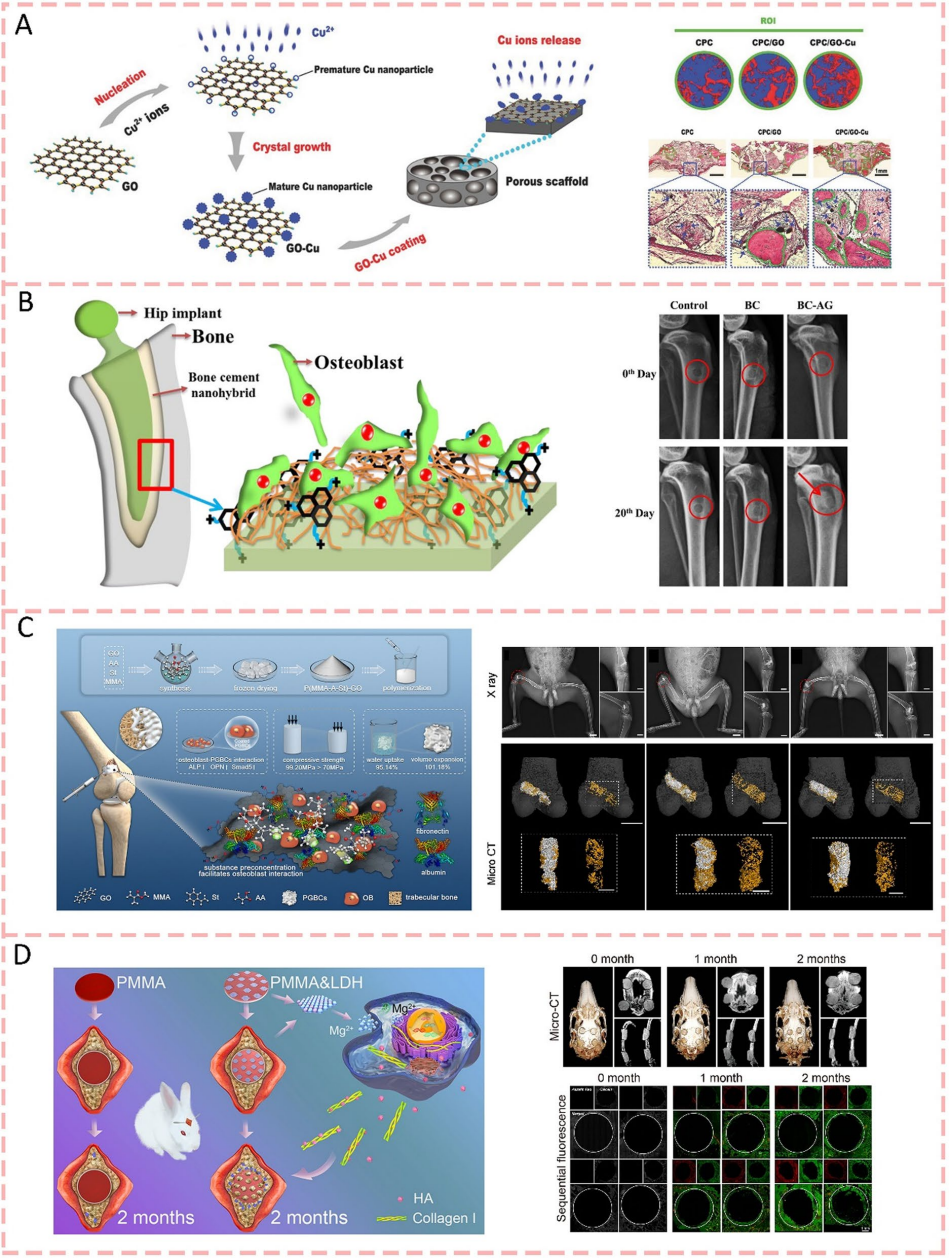
Graphene or LDH nanoparticles modified bone cements for bone regeneration. **A** Schematic illustrating graphene oxide-copper nanocomposite (GO-Cu) coated calcium phosphate cement for enhanced angiogenesis and osteogenesis [162]. Copyright 2016, WILEY–VCH Verlag GmbH. **B** The amine-functionalized graphene modification PMMA bone cement effectively reduced cellular oxidative stress and promoted calcification [163]. Copyright 2017, American Chemical Society. **C** The hydrophilic graphene oxide optimized both the biomechanical performance and bioactivity of P(MMA-AA-St) bone cement [164]. Copyright 2022, Elsevier. **D** Layered double hydroxides actively participated in cellular signaling through the controlled release of magnesium ions and created a favorable microenvironment for bone formation through their unique layered structure [167]. Copyright 2021, American Chemical Society

Adding graphene to bone cements improves the mechanical properties of compounds [160]. In vitro experiments, graphene is reported to have osteoinductivity and a high potential for promoting osteogenesis and osseointegration and further repairs bone defects [161]. Zhang et al. (2016) [162] synthesized a new bone regeneration system by coating porous calcium phosphate cement with graphene oxide-copper nanocomposite (GO-Cu). Graphene oxide functioned as a multifunctional platform in this system by serving as both a carrier for controlled copper ion release and a bioactive interface that enhanced cell adhesion and osteogenic differentiation, while its unique surface chemistry enabled uniform coating of the CPC scaffold. It improved the adhesion and proliferation of BMSC and osteogenic differentiation. When implanted in rat calvarial defects, the GO-Cu coated scaffolds showed superior angiogenic and osteogenic effects compared to unmodified CPC (Fig. 10a). In 2017, an investigation explored the modification of PMMA bone cement using graphene, graphene oxide, and amine-functionalized graphene as fillers. From an osteogenic perspective, the amine-functionalized graphene composite demonstrated exceptional osteointegration capabilities while maintaining minimal cytotoxicity. The material exhibited remarkable biological performance when tested in vivo, promoting calcification within 20 days of rabbit implantation and effectively reducing cellular oxidative stress. A notable feature of these graphene derivatives was their apparent ability to establish natural bonds with bone tissue, which enhanced both the bioactivity and long-term stability of prosthetic implants (Fig. 10b) [163]. The hydrophilic graphene oxide, leveraging its excellent biomechanical properties and osteogenic capacity, successfully optimized both the biomechanical performance and bioactivity of P(MMA-AA-St) bone cement. In vivo studies conducted using rabbit femoral condyle defect models demonstrated enhanced bone-material contact and superior osteogenic capabilities of the modified cement (Fig. 10c) [164].

Layered double hydroxides (LDHs) are two-dimensional nanomaterials composed of edge-sharing MO6 octahedral host layers and negatively charged interlayer anions [165, 166]. These materials possess excellent thermal insulation properties and good biocompatibility. In Wang et al.'s study (2021) [167], they demonstrated that LDH modification of PMMA bone cement resulted in several advantageous changes. LDH decreased the maximum polymerization temperature by 7.0 °C, which helped protect surrounding osteoblast-related cells from thermal damage. Although the mechanical properties slightly decreased after LDH modification, this reduction actually helped alleviate stress-shielding osteolysis and indirectly promoted osseointegration. Unlike other inorganic nanomaterials such as hydroxyapatite which mainly provides mechanical support, LDH actively participates in cellular signaling through controlled release of magnesium ions and creates a favorable microenvironment for bone formation through its unique layered structure (Fig. 10d).

### Polymer nanomaterials

**Fig. 11:**
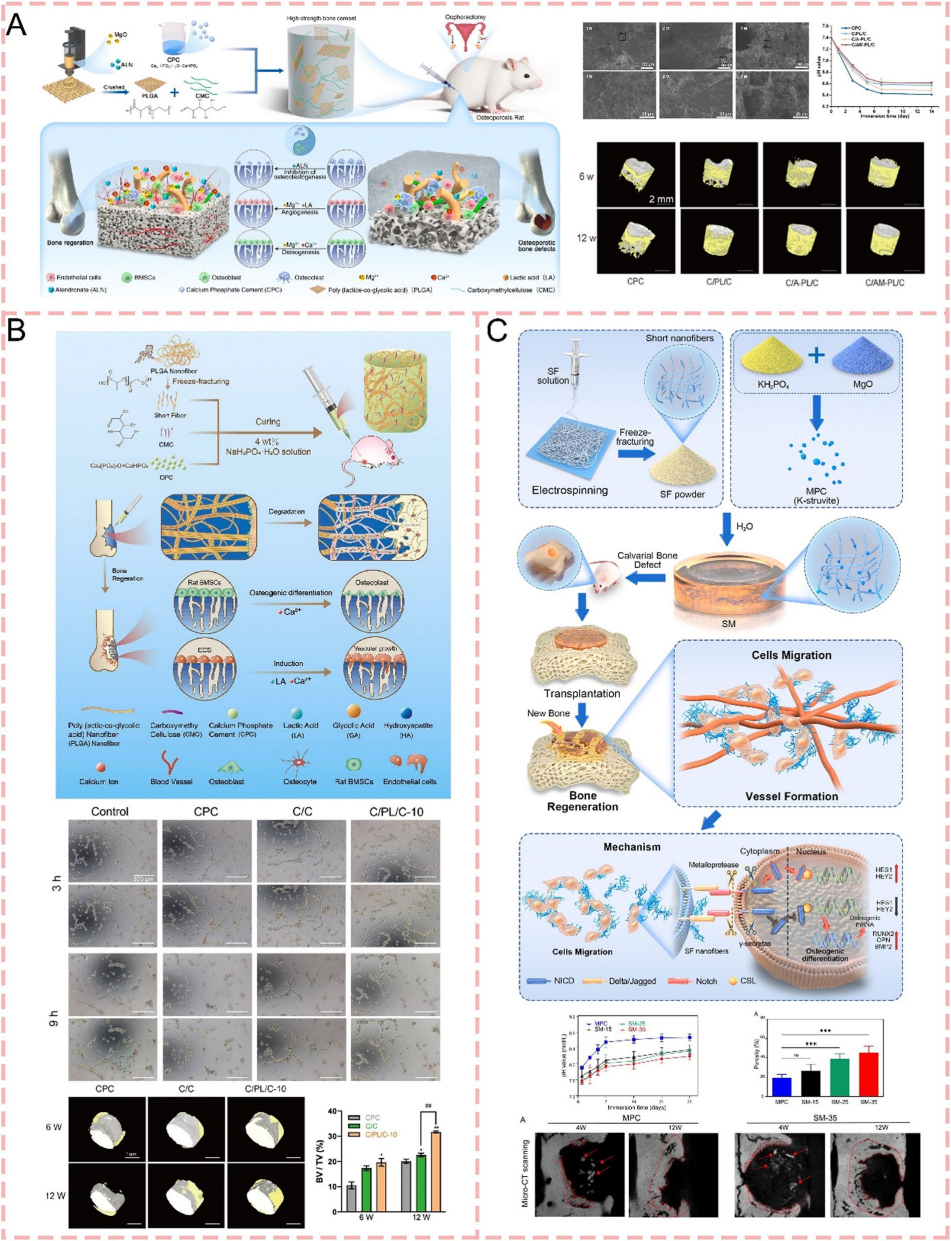
Polymer nanomaterials modified bone cements for bone regeneration. **A** Schematic illustrating calcium phosphate cement incorporated with PLGA nanofibers for enhanced angiogenesis, osteogenesis, and mechanical properties [168]. Copyright 2024, Elsevier. **B** Schematic showing calcium phosphate cement modified with short PLGA nanofibers to achieve dynamically controlled biodegradation, creating essential space for cell migration and bone ingrowth [169]. Copyright 2023, Elsevier. **C** Magnesium phosphate cement modified with electrospun silk fibroin nanofibers to enhance oxygen/nutrient infiltration and cell ingrowth while neutralizing excess magnesium oxide-induced alkalinity [42]. Copyright 2024, Elsevier

As mentioned earlier, PLGA is a primary synthetic polymer for bone cement modification. PLGA nanofibers, as a nanoscale morphology of PLGA materials, when used to modify bone cement, can achieve higher strength and injectability, and even carry drugs for bone regeneration treatment. According to Huang et al. [168], the incorporation of PLGA nanofibers effectively reduces CPC brittleness and improves its mechanical properties. As PLGA nanofibers gradually degrade, they generate interconnected pores that facilitate cell migration and bone ingrowth, enabling dynamically controllable biodegradability. The degradation process also releases bioactive components and creates a weakly acidic microenvironment, which synergistically promotes vascularization and bone regeneration. When combined with carboxymethyl cellulose, this PLGA nanofiber-modified CPC system demonstrates excellent injectability and anti-washout properties, making it particularly suitable for bone tissue engineering applications (Fig. 11a). Similarly, Cai et al. [169] developed an advanced bone cement system by incorporating short PLGA nanofibers into CPC. This modification endowed the cement with dynamically controllable biodegradability, creating essential space for cell migration and bone ingrowth through gradual fiber degradation. The lactic acid released during PLGA degradation further enhanced the system's performance by promoting vascularization, thereby establishing an integrated approach for effective bone regeneration (Fig. 11b). Through innovative biomaterial engineering approaches, Chen et al. [42] incorporated electrospun silk fibroin (SF) nanofibers with MPC. The resulting composite scaffold exhibited multiple enhanced properties, with its hierarchical structure enabling rapid oxygen and nutrient infiltration while promoting improved cell ingrowth. Additionally, the SF nanofibers strengthened the mechanical properties of MPC and effectively neutralized the highly alkaline environment generated by excess magnesium oxide, creating an optimized microenvironment for bone regeneration (Fig. 11c).

## Modified bone cements-mediated regulation of microenvironments to enhance bone regeneration

Drawing upon the distinct features of the bone microenvironment, this section reviews strategies for modulating the microenvironments with biomaterials-modified cements across different bone repair phases. During the initial period following bone trauma or loss, the primary focus is attenuating inflammatory responses and mitigating oxidative damage to preserve adjacent tissue integrity. In the bone regeneration phase, engineered biomaterials facilitate new vessel formation and stimulate bone-forming activities to expedite structural reconstruction. The mechanisms and biomaterials used in these strategies are summarized in Table 5. The following sections elaborate on these biomaterial-based strategies for orchestrating the microenvironmental conditions to optimize bone repair outcomes.

**Table 5:** Microenvironment modulation mechanisms by different biomaterials to enhance bone regeneration in different phases

Strategy	Cement	Modification method	Model	Simulated diseases	Mechanism	Refs.
Reduction of inflammation	CPC	Magnesium	In vitro	Bone substitute implantation	MCPC reduces macrophage M1 polarization to attenuate inflammation	[93]
	MPC	rhBMP2 + cement scaffold	SD rats	Bone substitute implantation	Mg ions reduce inflammation through macrophage regulation to enhance osteogenesis	[182]
	CS/CC/DCPA	Mg-MOF	In vitro	Non-load-bearing bone defects	Mg-MOF modulates macrophage M1/M2 polarization	[183]
	CPC	Zinc-doped calcium silicate	New Zealand white rabbits	Distal femoral bone defect	Zn-CS/CPC promoted the recruitment of macrophages and enhanced M2 polarization while inhibiting M1 polarization	[184]
	CaSO4 bone cement	Sr/Cu-BSG	SD rats/New Zealand white rabbits	Femoral condylar defects	BSG cement reduces M1 macrophage polarization and inflammatory cytokines while promoting M2 phenotype	[188]
Promotion of anti-oxidation	CPC	Se	SD rats	Osteoporotic bone defects	Se-CPC upregulates SOD2/GPX1 antioxidant enzymes	[194]
	CPC	Se	SD rats	Osteoporotic bone defects	Se-CPC rescued mitochondrial functions through activation of the GPx1-mediated antioxidant pathway	[195]
	PMMA	Amino graphene	Rabbits	Failure of the prosthesis	AG exerts antioxidant effects through free radical scavenging	[163]
	CPC	Fullerenol	In vitro	Bone reconstruction	The antioxidant activity of Ful not only protected cellular viability but also promoted osteogenic differentiation	[197]
Bone homeostasis maintenance	PMMA	TBB initiator	In vitro	Local osteolysis at the cement–bone interface	PMMA-TBB enhances polymerization efficiency and reduces monomer toxicity	[203]
	CPC	Solid lipid microparticles + ALN	In vitro	Severe bone turnover inhibition	Solid lipid microparticles deliver alendronate to promote osteogenesis while inhibiting osteoclastogenesis	[204]
	PMMA	Borosilicate Glass	SD rats/Goats	Tibia defects/Vertebral defect	BSG provides an alkaline microenvironment that spontaneously balanced the activities between osteoclasts and osteoblasts	[206]
	CPC	PEGs + ALN	SD rats	Osteoporotic bone defects	CPC buffers pH while ALN inhibits osteoclasts and Ca2 + promotes osteoblast differentiation	[43]
Prevascularization	CPC	Chitosan + Arg-Gly-Asp (RGD)	In vitro	Large skeletal defects	RGD-modified CPCs promote rapid vascular integration	[212]
	CPC	hPDLSCs + hUVECs	In vitro	Large skeletal defects	hPDLSCs support vessel formation through angiogenic factors while hUVECs enhance osteogenesis via paracrine signaling	[213]
	β-TCP scaffolds	In vivo bioreactors for prevascularization	SD rats	Tibia bone defect	In-vivo prevascularization in muscle pouch promotes vessel network formation within β-TCP scaffolds	[214]
Material-cell interactions	CPC	Calcium silicate + rhBMP-2	New Zealand rabbits	Femoral defects	Synergistic effects between calcium silicate and rhBMP-2 in promoting osteogenesis	[219]
	CPC	rhBMP2 + BMSC	Nude mice	Oral and maxillofacial defects	rhBMP2 enhances BMSCs proliferation and osteogenic differentiation through sustained release from CPC scaffolds	[220]
	CPC	chondroitin sulfate + PDA + rhBMP-2	SD rats	Bone tissue repair	CS enhances rhBMP-2 bioactivity by promoting BMPR expression and binding	[221]

### The early phase of bone trauma or loss

#### Reduction of inflammation

Inflammatory response serves as a fundamental mediator in bone healing dynamics. Yet, its impact presents a delicate balance: both insufficient and excessive inflammatory states, along with persistent chronic inflammation, can significantly impair the healing trajectory [170, 171]. The successful resolution of inflammatory processes during osseous repair relies heavily on intricate cellular cross-talk between immune components and various cell populations within the bone microenvironment, particularly multipotent mesenchymal stromal/stem cells (MSC) [172]. Consequently, A comprehensive approach to designing biomaterial-based bone cements must integrate direct osteogenic modulation alongside precise control of local inflammatory responses, ultimately fostering an optimal osteoimmune microenvironment that facilitates healing. Introducing biomaterials triggers a foreign body response, primarily mediated by macrophages for detecting and neutralizing various threats, ranging from pathogens and exogenous particles to senescent cells, abnormal stromal elements, and malignant cells [173]. Nevertheless, an excessive inflammatory reaction to biomaterial implantation may impede osteogenesis. Pro-inflammatory M1 phenotype macrophages release numerous inflammatory mediators that stimulate osteoclast formation and enhance their resorptive activity, ultimately promoting bone degradation [174]. In contrast, M2 macrophages produce beneficial factors such as VEGF, TGF-β1, and bone morphogenetic protein-2 (BMP-2), which facilitate angiogenesis and new bone formation [175]. The dynamic balance between these macrophage phenotypes fundamentally shapes the inflammatory bone microenvironment, orchestrating the intricate interplay between inflammation resolution and skeletal tissue regeneration, thereby playing a pivotal role in determining the success of biomaterial-mediated bone repair.

**Fig. 12:**
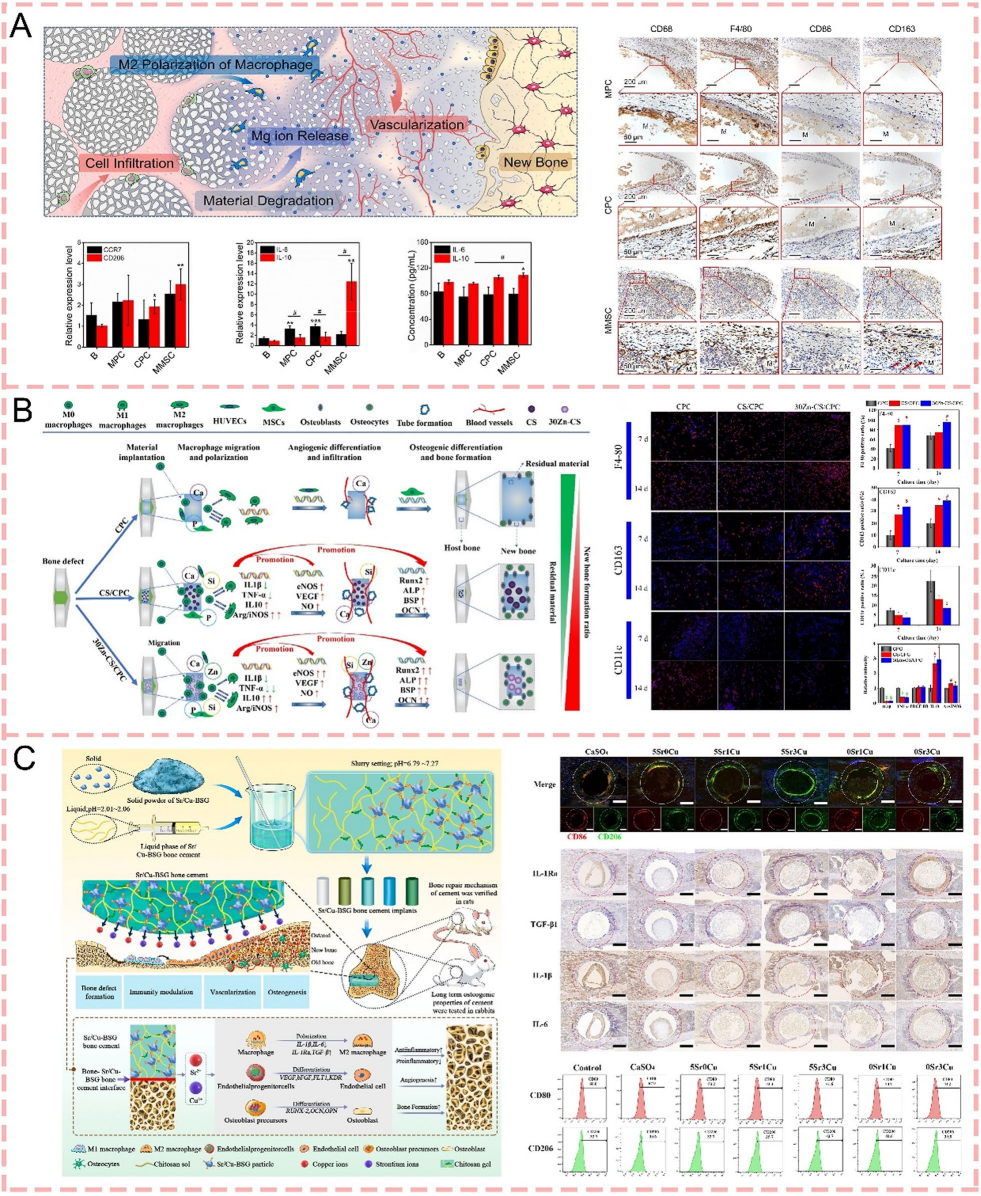
Modulating macrophage polarization to reduce microenvironmental inflammation. **A** Schematic illustrating magnesium-containing microsphere cement that modulates immune microenvironment through macrophage M2 polarization and IL-10 upregulation, promoting vascularization and bone formation [182]. Copyright 2021, Elsevier. **B** Schematic showing zinc-modified calcium silicate/calcium phosphate cement that selectively regulates macrophage phenotypes by promoting M2 while suppressing M1 polarization, leading to enhanced angiogenesis and osteogenesis [184]. Copyright 2022, Elsevier. **C** Schematic showing strontium/copper-doped borosilicate glass cement that modulates inflammatory gene expression in macrophages, upregulating IL-1Ra and TGF-β1 while downregulating IL-1β and IL-6 [188]. Copyright 2023, Elsevier

As a crucial physiological element, magnesium plays vital roles in various biological processes, from hormonal regulation to immune function and skeletal development [176–179]. Research has demonstrated that bone implants incorporating magnesium, exemplified by magnesium-based metals with β-TCP coating and hydroxyapatite scaffolds modified with MgSiO3, effectively modulate macrophage polarization toward an M2 phenotype and enhance bone regeneration [180, 181]. Wang et al. [93] applied magnesium-doped calcium phosphate cement to promote osteogenesis and angiogenesis by modulating macrophages toward M2 polarization and improving the secretion of regenerative cytokines. These observations highlight magnesium’s significant role in mediating favorable implant-host interactions during bone repair. Tan et al. [182] developed an injectable bone cement based on magnesium-containing microspheres (MMSC) that demonstrates enhanced osteogenesis through immunomodulatory effects. The released magnesium ions from MMSC effectively modulated the immune microenvironment by promoting the polarization of macrophages toward the anti-inflammatory M2 phenotype and upregulating anti-inflammatory cytokine IL-10 expression. This immunomodulatory effect subsequently facilitated vascularization and new bone formation. The synergistic effects of magnesium ion-mediated immunomodulation and the scaffold's structural advantages resulted in significantly improved bone regeneration outcomes, providing a promising strategy for bone defect repair through immunoregulation-enhanced osteogenesis (Fig. 12a). A novel bone cement system was developed by incorporating magnesium gallate metal–organic frameworks (Mg-MOF) into a tri-component matrix of calcium sulfate, calcium citrate, and dicalcium hydrogen phosphate anhydrous (CS/CC/DCPA). The integration of Mg-MOF bone cement demonstrated remarkable immunomodulatory capabilities by regulating macrophage polarization and modulating inflammatory factors [183]. The incorporation of zinc-modified calcium silicate into calcium phosphate cement generated a multifunctional biomaterial system with enhanced biological performance. This composite demonstrated superior immunomodulatory effects by facilitating macrophage recruitment and selectively promoting anti-inflammatory M2 phenotype while suppressing pro-inflammatory M1 polarization. The optimized immune microenvironment subsequently led to improved angiogenic responses in the early stage and enhanced bone regeneration capacity, suggesting a promising strategy for bone tissue engineering through zinc-mediated immunoregulation and osteogenesis coupling [184], as shown in Fig. 12b.

BGs can be doped with various elements, such as strontium, iron, copper, lithium, and manganese, to achieve specific biological effects, including immunomodulation, angiogenesis, and osteogenesis [185]. Zhang et al. [186] demonstrated that strontium-substituted bioactive glass (Sr-SBG) promoted macrophage polarization toward the anti-inflammatory M2 phenotype, thereby enhancing osteogenesis. Similarly, Lin et al. [187] reported that copper-incorporated bioactive glass–ceramics (Cu-BGC) facilitated cartilage regeneration by suppressing inflammation and inducing M2 macrophage polarization. Additionally, Li et al. (2023) [188] developed a bone cement system combining strontium- and copper-doped borosilicate glass (Sr/Cu-BSG) with a chitosan-based liquid phase to enhance bone healing. In vitro experiments revealed that the controlled release of Sr and Cu ions increased the expression of anti-inflammatory genes (IL-1Ra and TGF-β1) while simultaneously decreasing the expression of pro-inflammatory genes (IL-1β and IL-6) in macrophages within three days, demonstrating its immunoregulatory potential (Fig. 12c).

#### Promotion of anti-oxidation

**Fig. 13:**
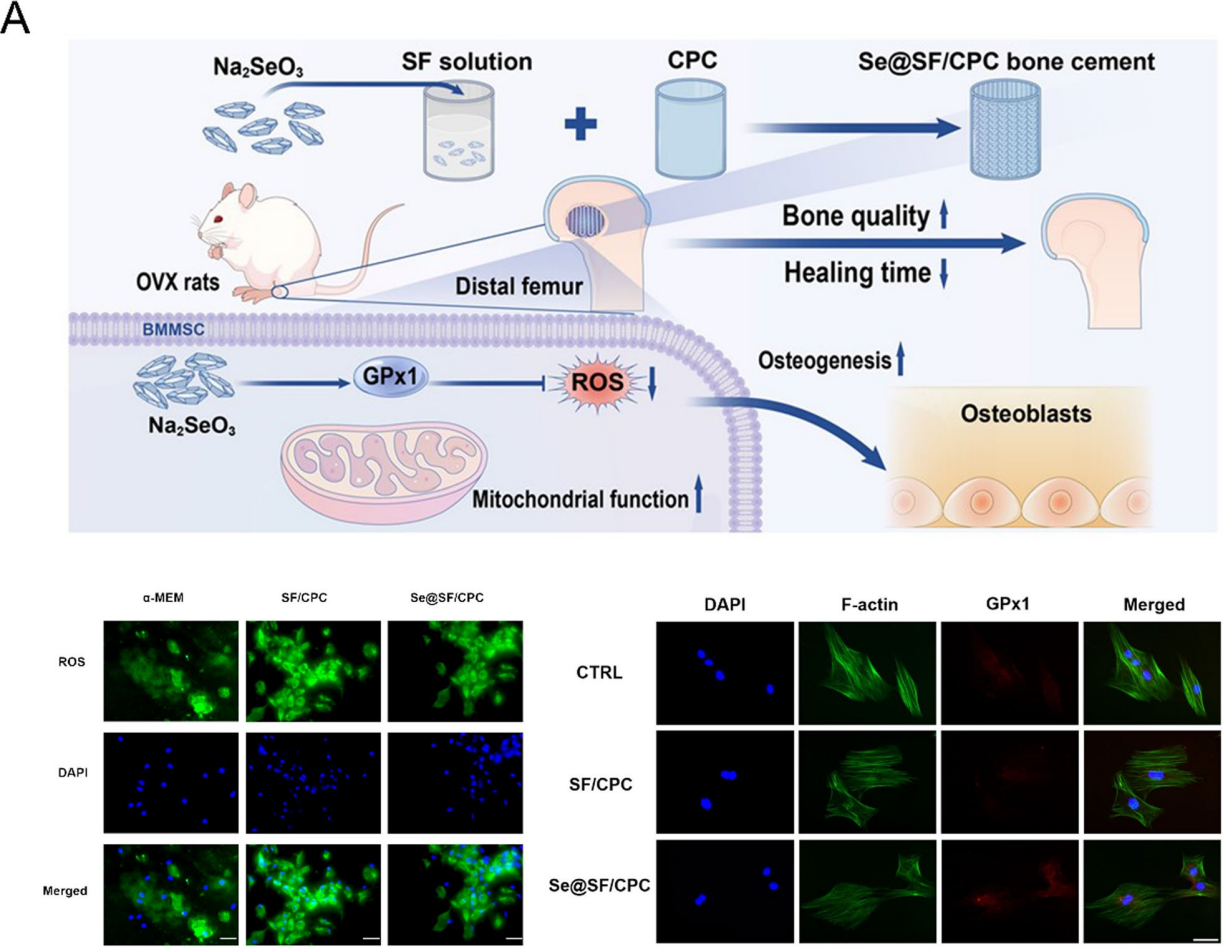
Promote antioxidant effects in the microenvironment. **A** Schematic depicting selenium-incorporated CPC that reduces ROS accumulation and restores mitochondrial function in osteoporotic BMSCs through GPx1 activation, enhancing osteogenic differentiation [195]. Copyright 2023, Oxford University Press

Previous research demonstrates that excessive reactive oxygen species (ROS) production induces cellular oxidative stress, leading to apoptosis in various bone-related cells, including mesenchymal stem cells, osteoblasts, and osteocytes [189, 190]. Furthermore, elevated ROS levels have been shown to impair the osteogenic differentiation capacity of stem and preosteoblast cells [191, 192]. Antioxidants like Trolox and selenium have been extensively investigated in bone tissue engineering and have been shown to enhance osteogenic differentiation while reducing oxidative stress in scaffold-based systems [193]. While these antioxidants have proven effective at scavenging reactive oxygen species when incorporated into brushite CPCs, their influence on osteogenic differentiation in cement-based systems remains unexplored. The study by Li et al. [194] demonstrated that selenium-modified calcium phosphate cement (Se-CPC) enhanced bone regeneration in osteoporotic defects through its antioxidant properties. By incorporating selenium into CPC scaffolds, the material significantly upregulated antioxidant enzymes SOD2 and GPX1 while reducing CAT expression, thereby mitigating local oxidative stress. This antioxidant effect improved bone mineralization, increased bone volume, and enhanced microarchitecture compared to conventional CPC, suggesting Se-CPC as a promising therapeutic strategy for treating osteoporotic bone defects. Another study also showed that the incorporation of selenium effectively reduces ROS accumulation and restores mitochondrial functions in osteoporotic bone marrow mesenchymal stem cells via activation of the GPx1 antioxidant pathway. This novel approach not only improves the osteogenic differentiation of BMSCs but also accelerates bone regeneration in ovariectomized rats, offering a promising therapeutic strategy for treating osteoporotic bone defects [195], as shown in Fig. 13a. Fullerenol (Ful), with its distinctive physicochemical properties, represents an advanced carbon-based material that exhibits exceptional reactive oxygen species neutralizing capabilities [196]. Duru et al. [197] presents a novel approach to enhance CPCs by incorporating Fullerenol, a water-soluble C60 fullerene derivative with potent antioxidant properties. The Ful-modified CPCs demonstrated significant ROS scavenging capacity, particularly at concentrations of 0.02 and 0.04 wt v%1. The antioxidant activity of Ful not only protected cellular viability but also promoted osteogenic differentiation, as evidenced by increased alkaline phosphatase activity and enhanced expression of runt-related transcription factor 2 in MC3 T3-E1 preosteoblast cells. Importantly, this modification did not compromise the cement's physical characteristics while improving its biological performance.

### The bone regeneration phase

#### The balance of osteoblasts and osteoclasts

**Fig. 14:**
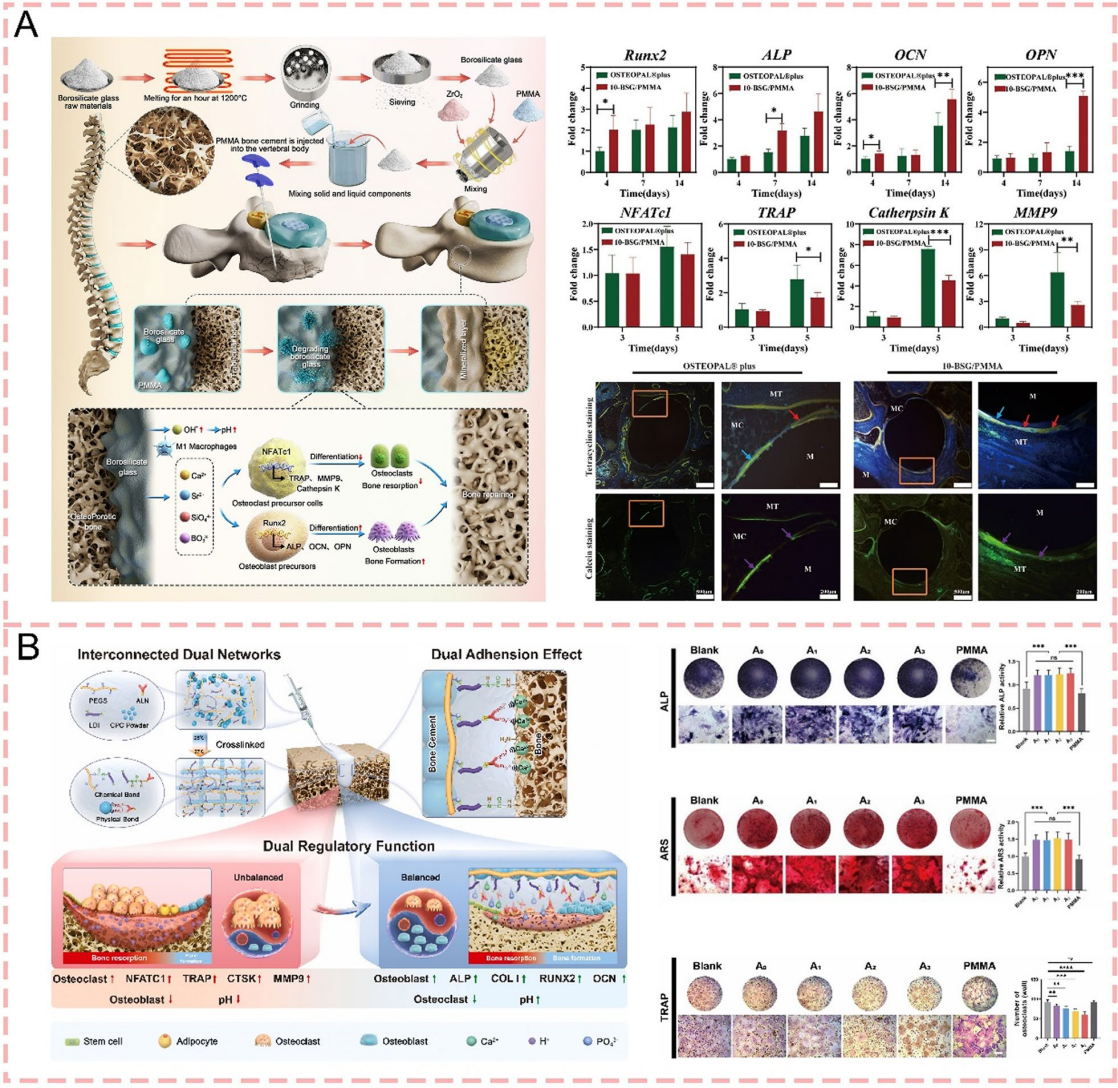
Adjusting the osteoblast-osteoclast balance in the microenvironment. **A** Borosilicate glass-modified PMMA bone cement generates an alkaline microenvironment that spontaneously modulates the balance between osteoclast and osteoblast activities [206]. Copyright 2022, American Chemical Society. **B** An injectable double-crosslinked bone cement system (PEGS/CPC@ALN) that effectively restored the balance between bone formation and resorption and promoted osteoregeneration in osteoporotic conditions [43]. Copyright 2025, Elsevier

Bone homeostasis disruption, particularly through excessive osteoclast activity, underlies the pathogenesis of osteoporosis [198, 199]. This imbalance manifests as compromised bone architecture, diminished mechanical integrity, and heightened fracture risk [200, 201]. The altered metabolic environment shifts mesenchymal stem cell fate towards adipogenesis while simultaneously accelerating the catabolism of newly synthesized bone matrix [202]. This pathological state severely impairs the bone's intrinsic healing capacity by preventing effective bridging of skeletal defects, highlighting the critical importance of maintaining proper osteoblast-osteoclast equilibrium for optimal bone regeneration. Tri-n-butylborane (TBB) initiator modified PMMA bone cement exhibits enhanced biocompatibility compared to conventional benzoyl peroxide (BPO)-initiated PMMA by promoting osteoblast survival, attachment, proliferation, and differentiation while simultaneously reducing osteoclastogenesis. The improved bone homeostasis maintenance through this dual regulation of increased osteoblastic activity and decreased osteoclast formation [203]. Alendronate, as a nitrogen-containing bisphosphonate, effectively decreases bone turnover and prevents bone loss by inhibiting osteoclast activity, making it a potent antiresorptive agent for treating osteolytic diseases [204]. Dolci et al. [205] demonstrated that alendronate-loaded CPC effectively modulates bone homeostasis by simultaneously promoting osteoblast viability and differentiation while inhibiting osteoclast activity. Through the incorporation of solid lipid microparticles as drug carriers, the system achieved sustained release of alendronate, resulting in enhanced osteogenic properties and controlled bone resorption. This dual-action mechanism helps maintain the delicate balance between bone formation and resorption, potentially improving bone regeneration outcomes in clinical applications. The study by Zhang et al. (2022) [206] showed that borosilicate glass-modified PMMA bone cement generates an alkaline microenvironment that spontaneously modulates the balance between osteoclasts and osteoblasts activities. The released trace elements from BSG enhance osteogenesis, while the alkaline conditions inhibit excessive osteoclastogenesis, thereby promoting a favorable bone homeostasis at the implant-host tissue interface. This dual-functional approach effectively addresses the biological limitations of traditional PMMA cement by facilitating osseointegration and strengthening the bone-implant interface, as shown in Fig. 14a. Recently, Zhao et al. [43] presented an injectable double-crosslinked bone cement system (PEGS/CPC@ALN) that effectively regulates bone homeostasis and promotes osteoregeneration in osteoporotic conditions. The system combines PEG, CPC, and ALN to create a dual-network structure that not only provides mechanical support but also actively modulates the pathological bone microenvironment. The cement system maintains bone homeostasis via multiple synergistic mechanisms. The buffering effect of CPC works to improve the acidic microenvironment, while controlled ALN release serves to inhibit excessive osteoclast activity. Additionally, the sustained release of calcium ions stimulates osteoblast differentiation. This comprehensive approach effectively restores the balance between bone formation and resorption, creating an optimal microenvironment for bone regeneration in osteoporotic conditions (Fig. 14b).

#### Prevascularization

**Fig. 15:**
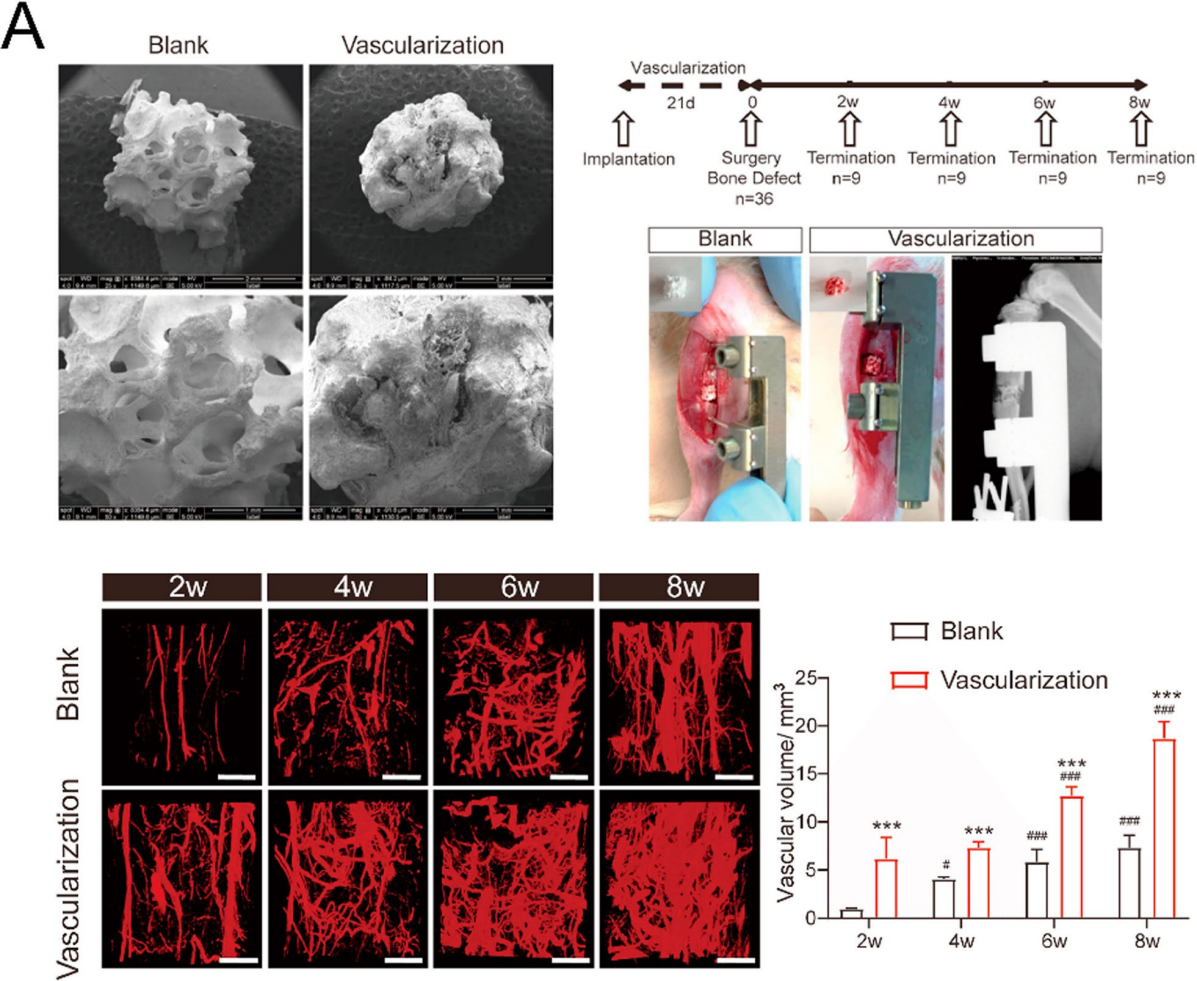
Prevascularization for bone regeneration. **A** Schematic illustration of muscle-based in vivo prevascularization strategy using β-TCP scaffolds, demonstrating enhanced vascularization and bone regeneration through upregulated BMP2 and VEGF expression [214]. Copyright 2022, Elsevier

The success of bone regeneration is fundamentally dependent on adequate vascularization, as insufficient blood vessel penetration into bone graft centers significantly impairs bone healing and consolidation [207, 208]. To address this critical challenge, in vivo prevascularization has emerged as a promising approach for enhancing bone tissue engineering outcomes [209]. This technique involves the establishment of functional vascular networks prior to graft implantation, which substantially accelerates host vessel integration and subsequent tissue regeneration. Several in vivo prevascularization methods have been developed, such as incorporating periosteal flaps, establishing arteriovenous loops, and integrating vascular bundles within or surrounding the grafts [210, 211]. These strategies effectively promote the formation of a robust vascular network, ultimately supporting successful bone regeneration. As early as 2014, Chen et al. [212] developed a novel prevascularized CPC scaffold by incorporating RGD peptides and co-culturing HUVEC and human osteoblasts (HOB). The RGD-modified macroporous CPC significantly enhanced the formation of microcapillary-like structures, osteogenic differentiation, and bone mineral synthesis compared to unmodified CPC. After 42 days of culture, the cumulative vessel length on RGD-modified CPC was 1.69-fold higher than the control. The enhanced prevascularization and osteogenic capacity suggest this biomaterial system's potential for improved bone regeneration by facilitating rapid vascular integration upon implantation in dental and craniofacial applications. Furthermore, the addition of perivascular cells (pericytes) in a tri-culture system substantially enhanced the stability and maturation of the primary vascular network, leading to improved blood vessel formation and bone regeneration in vivo. Meanwhile, Zhao et al. [213] first demonstrated that the co-culture of hUVECs and hPDLSCs on CPC scaffolds achieved excellent osteogenic and angiogenic capabilities in vitro, successfully generating prevascularized networks for potential tissue engineering applications. Recently, Xu et al. [214] developed a novel in vivo prevascularization strategy using β-TCP scaffolds to enhance bone regeneration. By implanting β-TCP scaffolds in a muscle pouch adjacent to the tibia defect site for 3 weeks before transplantation, they successfully achieved enhanced vascularization and bone formation compared to non-prevascularized scaffolds. This approach demonstrates several advantages compared to previous strategies by utilizing β-TCP's excellent osteoconductivity and biodegradability, avoiding the complexity of in vitro prevascularization methods, and achieving superior integration with host vasculature. Their findings showed significantly improved angiogenesis and osteogenesis through upregulated expression of BMP2 and VEGF, providing a promising and clinically translatable solution for large bone defect repair (Fig. 15a).

#### Material-cell interactions

**Fig. 16:**
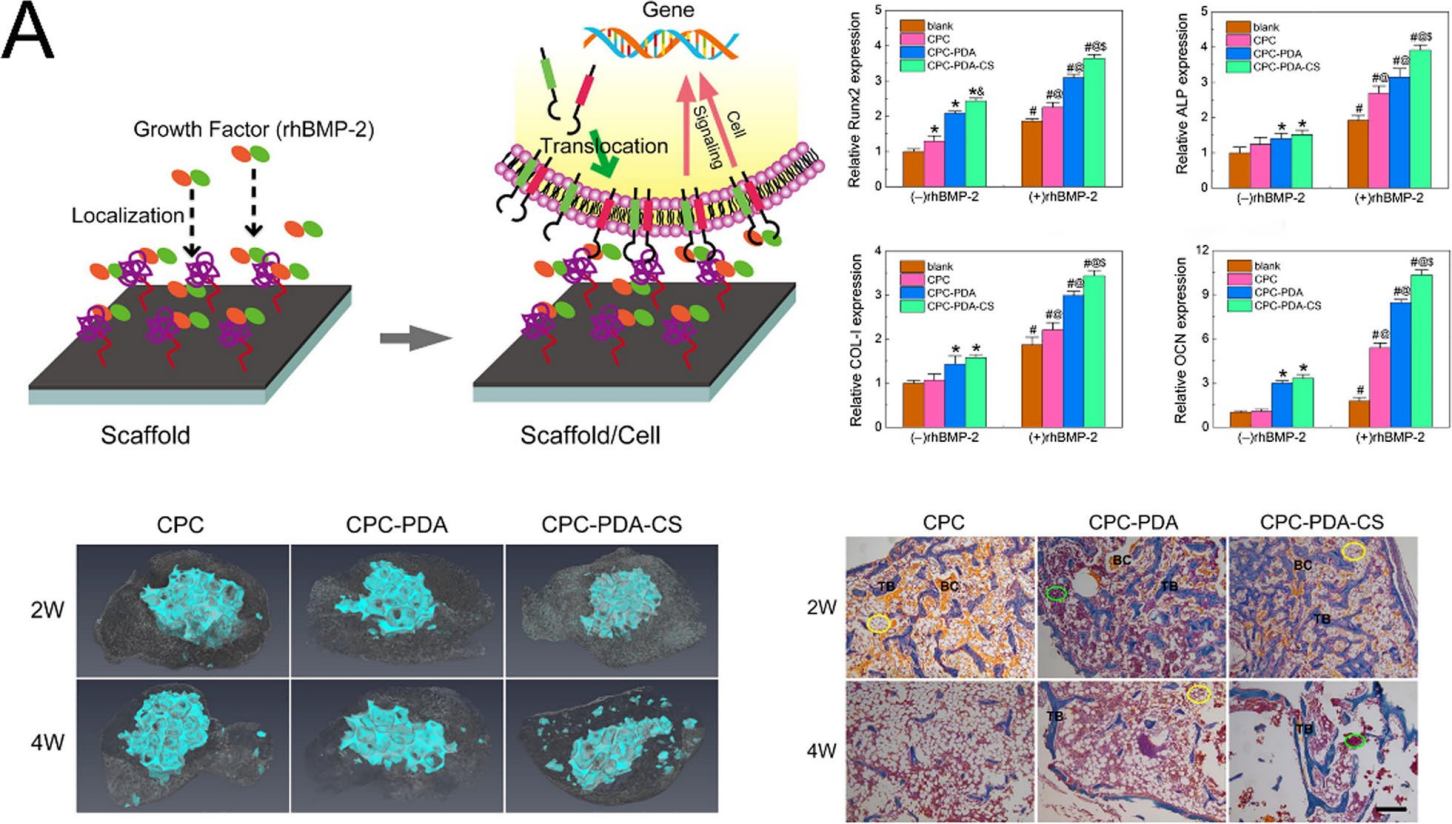
Bone cell surface receptor-targeted modification in bone microenvironment. **A** Schematic showing CS-functionalized CPC scaffolds that enhance BMP receptor-ligand recognition and surface translocation, particularly BMPR-IA, activating Smad1/5/8 and ERK1/2 pathways for sustained osteogenesis [221]. Copyright 2022, Elsevier

In the bone microenvironment, the biological interactions between materials and bone cells are fundamentally important, particularly through the mediation of surface physicochemical properties. [215, 216] These interactions involve complex cascades between immobilized proteins, extracellular matrices, and host cells. As a prominent example, rhBMP-2, a member of the TGF-β superfamily approved by both FDA and EMA [217, 218], serves as a crucial osteoinductive growth factor that significantly influences bone regeneration through these material-cell interactions. Zhang et al., through in vitro studies with C2 C12 cells and in vivo experiments, demonstrated that the CSPC/rhBMP-2 scaffold significantly enhanced osteogenic differentiation and bone regeneration compared to conventional CPC. Notably, the silicon ions released from CSPC helped maintain rhBMP-2’s bioactive conformation, enabling synergistic effects between calcium silicate and rhBMP-2 in promoting osteogenesis [219]. Luo et al. (2017) [220] also demonstrated that the combinations of rhBMP2-loaded CPC with allogeneic BMSCs promote new bone tissue formation. Recently, Huang et al. [221] developed an innovative approach to enhance bone regeneration by functionalizing CPC scaffolds with chondroitin sulfate (CS) for controlled delivery of rhBMP-2. Through specific non-covalent interactions between CS and rhBMP-2, the modified scaffolds demonstrated a superior ability to localize and sustain the bioactivity of rhBMP-2. Notably, the CS-functionalized CPC scaffolds significantly enhanced the expression and cellular surface translocation of BMP receptors (particularly BMPR-IA) and improved receptor-ligand recognition, leading to enhanced downstream signaling through the Smad1/5/8 and ERK1/2 pathways. This biomimetic approach resulted in more effective and sustained osteogenic stimulation both in vitro and in vivo, providing new insights into the design of growth factor delivery systems for bone tissue engineering applications (Fig. 16a).

## Conclusion and future perspectives

Impaired bone healing arising from neoplasms, infections, severe trauma, and osteoporotic fractures poses substantial healthcare challenges with profound socioeconomic implications [222]. Despite advances in medical science, the development of optimal therapeutic strategies remains elusive. Recent progress in biomaterial engineering has introduced innovative approaches to enhance bone cement properties through the incorporation of materials with special functions and unique structures. The pathophysiology of bone lesions involves complex alterations in the local microenvironment, characterized by an initial inflammatory response that potentially exacerbates bone resorption. This emerging understanding has led to increased focus on therapeutic strategies targeting microenvironmental modulation to promote bone regeneration. This review presents the first comprehensive analysis of contemporary materials used to modify bone cement to enhance bone regeneration, with particular emphasis on their capacity to modulate the bone microenvironment.

Bone cements serve multiple clinical functions, including void filling in cancellous bone, prosthetic fixation, and osteoporotic bone reinforcement [223]. The biomimetic approach to bone tissue engineering focuses on replicating the protein-mineral composite structure of natural bone to facilitate functional regeneration [224]. For example, while calcium phosphate cements have demonstrated promise due to their compositional similarity to native hydroxyapatite, their clinical application faces limitations including insufficient mechanical properties and prolonged setting kinetics [225, 226]. Recent studies demonstrate that strategic incorporation of biological molecules, such as hyaluronic acid, can enhance both the mechanical performance and osteogenic potential of CPCs through modulation of gene expression pathways [225]. The therapeutic potential of inorganic materials lies in their controlled ionic release mechanisms, which can orchestrate multiple aspects of tissue regeneration [227]. However, the clinical translation of ion-based therapeutics faces challenges related to spatial control and off-target effects. Biomaterial-based delivery systems offer a promising solution for localized ionic delivery to pathological bone sites. Complementarily, organic materials serve dual functions in bone cement modification: enhancing mechanical integrity while promoting biological processes including mineralization and osteoconduction. The integration of these materials into bone cements introduces multifunctional capabilities through their inherent biocompatibility and controlled degradation profiles.

The integration of nanotechnology into bone regeneration represents a significant paradigm shift in biomaterial engineering [228]. Nanomaterials have emerged as versatile modifiers for cementitious composites, offering enhanced mechanical properties, refined microstructural characteristics, and improved material longevity. For example, Fe3O4/GO nanocomposites enable controlled hyperthermia (43–46 °C) under magnetic stimulation for tumor cell elimination while preserving cement integrity, while iron oxide nanoparticles (IONPs) significantly enhance both mechanical properties and cellular responses, particularly improving human dental pulp stem cell adhesion and proliferation. The evolution of nanostructured materials has particularly revolutionized drug delivery strategies in bone cement applications [229]. Biodegradable polymers and high-molecular-weight carriers have formed the foundation for sophisticated nano-delivery systems [230]. A notable advancement came in 2017 with the successful incorporation of mesoporous silica nanoparticles into PMMA bone cement. The modification of cement systems with drug-loaded MSNs, such as sodium alendronate-bearing platforms, has demonstrated comprehensive improvements in mechanical strength, setting kinetics, and injectability while maintaining excellent biocompatibility and enhanced osteogenic potential. Further innovations include the development of PLGA nanofibers systems loaded with alendronate, specifically designed to optimize bone regeneration in osteoporotic conditions.

These advances in nanomaterial-modified bone cements demonstrate remarkable potential in enhancing mechanical properties and drug delivery capabilities. However, the success of bone regeneration ultimately depends on the complex interplay between biomaterials and the local tissue microenvironment. The bone microenvironment, comprising multiple cell types, extracellular matrix components, and signaling molecules, plays a pivotal role in determining therapeutic outcomes. Understanding and modulating this microenvironment has emerged as a crucial strategy for optimizing bone regeneration. Recent developments in material design have therefore focused on creating smart biomaterials that can actively respond to and regulate the local microenvironment, particularly addressing challenges such as inflammation, oxidative stress, and impaired vascularization.

Persistent inflammatory responses following skeletal trauma significantly impair osteogenic processes [231]. Early intervention to suppress inflammatory cell accumulation presents a crucial strategy for preventing bone resorption [232]. The therapeutic potential of macrophage phenotype modulation extends beyond anti-inflammatory effects to actively promote tissue regeneration [233]. A notable advancement in this field involves nanoparticles with negative zeta potential, which demonstrate dual functionality in microenvironment regulation by simultaneously influencing macrophage polarization and attenuating immune cell infiltration [234]. This discovery provides valuable insights for the rational design of drug-delivery nanoplatforms. In parallel with inflammation management, the modulation of oxidative stress emerges as a critical therapeutic target. Enhancement of endogenous antioxidant enzyme systems, coupled with controlled release of exogenous antioxidants, effectively mitigates elevated reactive oxygen species levels, facilitating tissue recovery [235]. The development of injectable antioxidant-loaded nanocarriers represents a promising direction for future research, offering the potential for rapid intervention and immediate protective effects in acute bone injury scenarios.

Over the past several years, research has increasingly highlighted the critical role of sensory innervation in maintaining skeletal homeostasis, offering novel perspectives for therapeutic interventions in bone disorders [236]. Neuropeptides secreted by sensory nerve fibers, particularly calcitonin gene-related peptide and substance P, have emerged as key mediators of osteogenesis in metabolically active bone regions [237]. The identification of semaphorins, beyond their classical role in axonal guidance, has revealed their significant contribution to bone homeostasis. Despite compelling evidence supporting neural involvement in bone repair, current therapeutic strategies have yet to fully incorporate innervation components. Research suggests that establishing neurovascularized networks within bone scaffolds could enhance tissue regeneration quality and accelerate healing processes [238]. This is supported by studies showing compromised bone repair, characterized by reduced sensory innervation, impaired vascularization, and delayed callus ossification, in models with inhibited NGF signaling [239, 240]. Recent findings indicate that magnesium malate-modified calcium phosphate cement influences osteogenesis through a complex interplay involving macrophages, dorsal root ganglia neurons, and osteoblasts via the Mg^2+^-PGE2-CGRP pathway. This mechanism has been further elucidated by Zhang's work, demonstrating magnesium ion-induced CGRP production in dorsal root ganglia and subsequent enhancement of osteogenic differentiation [241]. The emergence of magnesium phosphate cement as an environmentally friendly binding material with inherent osteogenic properties represents a significant advance in bone tissue engineering. In light of these findings, future research directions should focus on the strategic modification of MPC to modulate the bone microenvironment, with particular emphasis on sensory nerve regulation. The inherent magnesium ion release capability of MPC, coupled with its potential for drug loading and delivery, presents a promising platform for enhanced therapeutic interventions in bone regeneration.

Overall, the continual advancement in our comprehension of bone microenvironment mechanisms, coupled with innovations in biomaterial science, heralds a new era in therapeutic approaches. The evolution of biomaterial-modified bone cements presents unprecedented opportunities for precise microenvironmental modulation. As this field progresses, novel therapeutic targets and intervention strategies are likely to emerge, expanding the frontier of regenerative medicine. These developments signal a promising future where biomaterial-based therapies will significantly enhance bone regeneration and functional recovery, ultimately leading to superior clinical outcomes and improved quality of life for patients with skeletal disorders.

## Data Availability

No datasets were generated or analysed during the current study.
